# Exosome-mediated miR-144-3p promotes ferroptosis to inhibit osteosarcoma proliferation, migration, and invasion through regulating ZEB1

**DOI:** 10.1186/s12943-023-01804-z

**Published:** 2023-07-17

**Authors:** Mingyang Jiang, Yiji Jike, Kaicheng Liu, Fu Gan, Ke Zhang, Mingjing Xie, Junlei Zhang, Chuanliang Chen, Xiaochong Zou, Xiaohong Jiang, Yongheng Dai, Weikui Chen, Yue Qiu, Zhandong Bo

**Affiliations:** 1 Department of Bone and Joint Surgery, The First Affiliated Hospital of Guangxi Medical University, Nanning, Guangxi China; 2 Department of Urology Surgery, The Affiliated Hospital of Youjiang Medical University for Nationalities, Baise, China; 3 Department of Sports Medicine, Southern University of Science and Technology Hospital, Shenzhen, China; 4 Department of Trauma Orthopedic and Hand Surgery, The First Affiliated Hospital of Guangxi Medical University, Nanning, China; 5 Department of Radiology, The First Affiliated Hospital of Guangxi Medical University, Nanning, China

**Keywords:** Osteosarcoma, Exosome, miR-144-3p, ZEB1, Ferroptosis

## Abstract

**Background:**

Osteosarcoma (OS) is the most prevalent orthopedic malignancy with a dismal prognosis. The high iron absorption rate in OS cells of patients suggests that ferroptosis may be related to the progression of OS, but its potential molecular regulatory role is still unclear. Based on the ability to couple with exosomes for targeted delivery of signals, exosome-derived micro ribonucleic acids (miRNAs) can potentially serve as diagnostic biomarkers for OS.

**Methods:**

We identified ferroptosis-related miRNAs and messenger ribonucleic acids(mRNAs) in OS using bioinformatics analysis and performed survival analysis. Then we measured miRNA expression levels through exosome microarray sequencing, and used RT-qPCR and IHC to verify the expression level of miR-144-3p and ZEB1. Stable gene expression cell lines were fabricated for in vitro experiments. Cell viability, migration and invasion were determined by CCK-8 and transwell experiment. Use the corresponding reagent kit to detect GSH/GSSG ratio, Fe^2+^ level, MDA level and ROS level, and measure the expression levels of GPX4, ACSL4 and xCT through RT-qPCR and WB. We also constructed nude mice model for in vivo experiments. Finally, the stability of the miRNA/mRNA axis was verified through functional rescue experiments.

**Results:**

Low expression of miR-144-3p and high expression of ZEB1 in OS cell lines and tissues was observed. Overexpression of miR-144-3p can promote ferroptosis, reduce the survival ability of OS cells, and prevent the progression of OS. In addition, overexpression of miR-144-3p can downregulate the expression of ZEB1 in cell lines and nude mice. Knockdown of miR-144-3p has the opposite effect. The functional rescue experiment validated that miR-144-3p can regulate downstream ZEB1, and participates in the occurrence and development of OS by interfering with redox homeostasis and iron metabolism.

**Conclusions:**

MiR-144-3p can induce the occurrence of ferroptosis by negatively regulating the expression of ZEB1, thereby inhibiting the proliferation, migration, and invasion of OS cells.

**Graphical Abstract:**

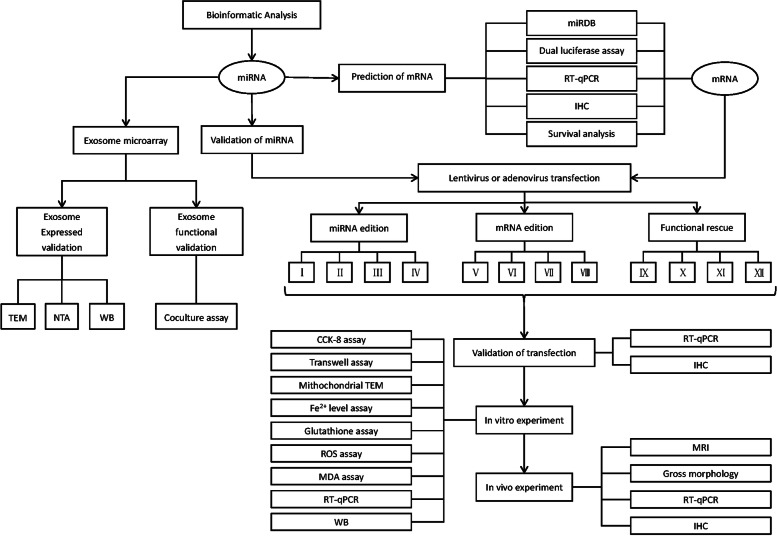

**Supplementary Information:**

The online version contains supplementary material available at 10.1186/s12943-023-01804-z.

## Introduction

Osteosarcoma (OS) is the most prevalent malignant bone neoplasm with common occurrence in children and adolescents [1], accounting for the largest proportion of orthopedic malignant tumors. Numerous studies have shown that 80% of OS cases were confirmed with local invasion and distant metastasis [2]. At present, the common treatment options for patients with OS are surgical resection, radiotherapy, and chemotherapy. Through chemotherapy, the 5-year survival rate and quality of life for patients were significantly improved, reaching approximately 60% [3]. However, drug resistance after chemotherapy and distant metastasis after surgery are still the substantial reasons and key factors leading to poor prognosis, treatment failure, and tumor recurrence. As a highly heterogeneous tumor, OS usually shows aberrant gene expression and molecular regulating mechanisms [4]. Research on targeted therapy for OS has also begun to emerge. For example, TP53 inhibitor NSC59984 can promote the death of OS cells by inducing the degradation of mutant p53 protein [5], and dual phosphoinositide 3-kinase (PI3K) mammalian target of rapamycin (mTOR) inhibitor NVP-BEZ235 can inhibit the proliferation of OS cells and improve prognosis [6]. However, because correlative therapeutic strategies are still in the experimental stage, many regimens have not been promoted for use in clinical treatment, and there is still a need for better methods for early prediction and treatment of OS. Therefore, it is imperative to elucidate the molecular mechanism of OS progression and identify a novel diagnostic signature, thereby exploring therapeutic strategies to improve the prognosis of OS.

Studies have indicated that micro ribonucleic acid (miRNA), a small single-stranded ribonucleic acid (RNA) transcribed from genes, is involved in the process of tumor pathophysiology and distant metastasis [7]. The miRNA is a single-stranded RNA [8] containing 18–25 noncoding nucleotides, can affect protein biosynthesis by regulating post-transcription, is involved in multiple aspects of cell biological activity regulation, such as cell proliferation, differentiation, and death, and performs a pivotal function in the formation, progression, and metastasis of malignant neoplasm [9]. Many miRNAs have been confirmed to have a specific expression in different tumors, and their expression is significantly different in normal tissues and tumor tissues. Through bioinformatics analysis, we found that miR-144-3p is significantly lower in OS cells.Among 13 different diseases, the level of miR-144 in plasma has been reduced [10, 11]. Zhao et al. found that miRNA-144-3p upregulation can inhibit the proliferation, invasion, and metastasis of OS by targeting a variety of messenger ribonucleic acids (mRNAs) (including TUG1, TAGLN, and EZH2) [12, 13], which indicates that miR-144-3p could potentially be a new target of OS therapy. However, the specific mechanism behind the effect on OS cells remains unclear. What is more interesting to us is that regulatory non-coding RNAs exist in all biological fluids related to protein complexes or are encapsulated in extracellular biological factors, such as microbubbles or exosomes.

The exosomes are tiny intercellular vesicles that carry lipids and proteins similar to those of the source cells. They are released from the cells to the extracellular microenvironment through exocytosis, interact with the receptor cells, trigger molecular release or signal transduction cascade induction, and finally lead to changes in cell activity or function [14]. Such characteristics enable them to be used by a variety of cancer cells for pathological transport to participate in the establishment of tumor microenvironment (TME) [15], promote tumor growth, promote cancer proliferation through tumor matrix interaction, and escape host destruction [16, 17]. Therefore, exosomes are also an important influencing factor for the occurrence and development of OS. But exosomes are a double-edged sword, playing an important role not only in tumor development, angiogenesis, and metastasis, but also in inhibiting tumor progression [18]. Cancer can be detected early by utilizing extracellular vesicles in the circulatory system. Therefore, exosomes derived from OS cells may become potential targets for their treatment, as they reflect the current state of the tumor [19]. However, the specific mechanisms of these RNA molecules and their expression in exosomes must be further elucidated to prevent the influence of unrelated proteins or harmful RNA molecules. More research is still needed to provide evidence to demonstrate the details and importance of extracellular vesicle mediated miRNA in the pathogenesis of OS, and to identify it as a core marker of cancer.

Ferroptosis, a novel model of cell death type first reported by Dixon et al. in 2012 [20], is a specific non-apoptotic death triggered by intracellular iron-dependent lipid peroxidation [21]. At present, emerging evidence has indicated that ferroptosis is widely implicated in the development of various malignant neoplasms [22]. In OS, Isani et al. first described iron-dependent and non-apoptotic ferroptosis-like cell death in OS cell line D-27, which can induce ferroptosis by inhibiting signal transducer and activator of transcription 3 (STAT3)/ nuclear factor erythroid 2-related factor 2 (Nrf2)/ glutathione peroxidase 4 (GPX4) signal [23]. It was revealed that OS can be more sensitive to drugs by using a ferroptosis inducer or STAT3 inhibitor. The above factors are closely related to OS, and there are few reports on the interaction between exosomes and ferroptosis in OS. The interaction between exosomes and ferroptosis in OS is likely to become a new therapeutic target for OS, but requires further research.

In this study, we used the exosomes as the vectors of miRNA-144-3p to determine the role of miR-144-3p overexpression and knockdown in the regulation of ferroptosis mechanism, to probe the relationship between miR-144-3p and the characteristics related to ferroptosis of OS, thereby studying its specific mechanism.

## Materials and methods

**Fig. 1:**
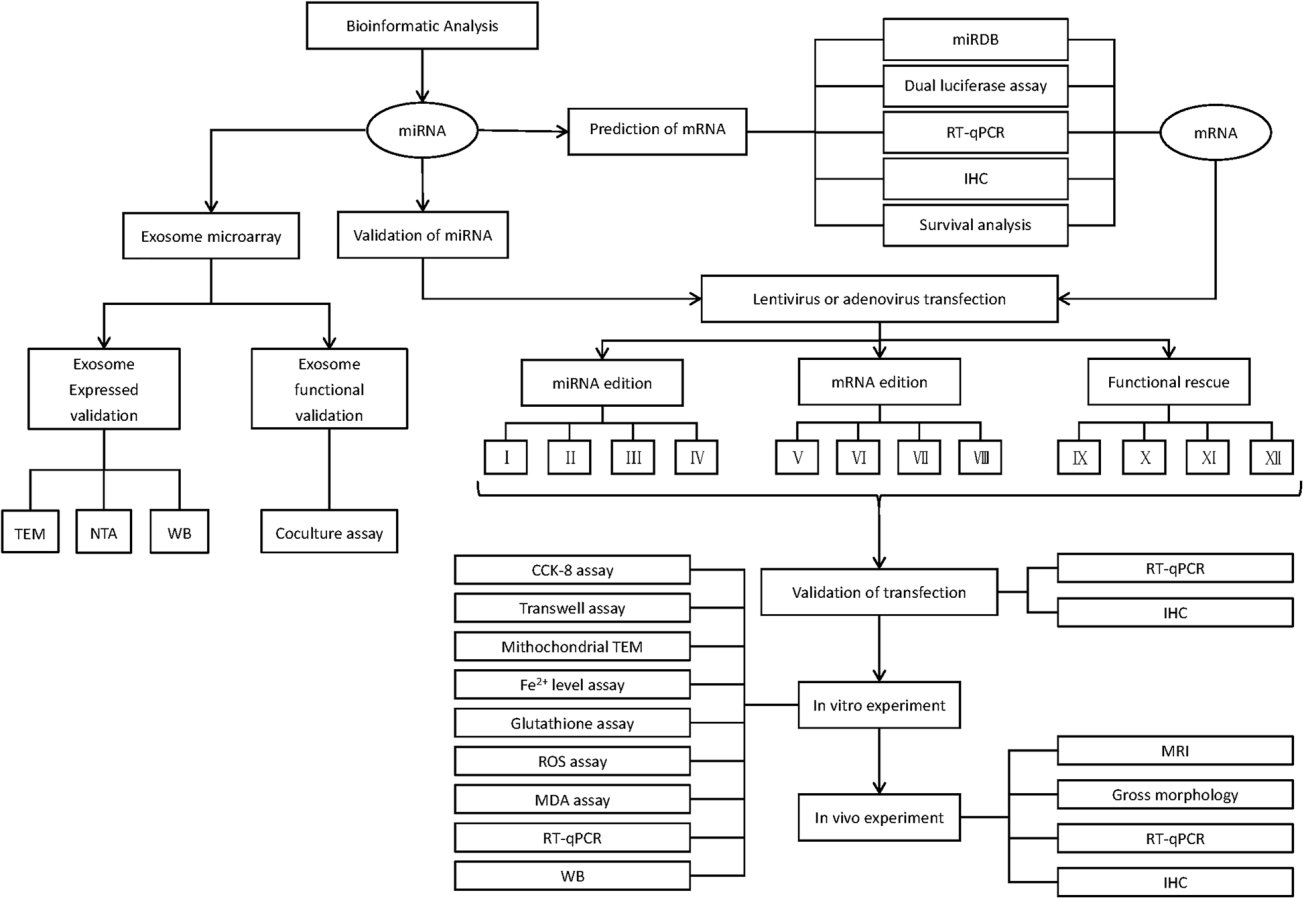
Graphical abstract. Groups of miR-144-3p/ZEB1 edition and functional rescue experiment. I: Normal control group of overexpress miR-144-3p. II: Overexpress miR-144-3p group. III: Normal control group of knockdown miR-144-3p. IV: Knockdown miR-144-3p group. V: Normal control group of overexpression ZEB1. VI: Overexpress ZEB1 group. VII: Normal control group of knockdown ZEB1. VIII: Knockdown ZEB1 group. IX: Normal control group of overexpress miR-144-3p & normal control group of overexpress ZEB1 group. X: Overexpress miR-144-3p & normal control group of overexpress ZEB1 group. XI: Normal control group of overexpress miR-144-3p & overexpress ZEB1 group. XII: Overexpress miR-144-3p & overexpress ZEB1 group

The whole relative procedures were performed as per the flow chart (Fig. 1).

### Cell culture

Five human OS cell lines (h143B, SW1353, MG-63, SaOS-2, U2OS) and corresponding normal osteoblastic cell lines (HOB) were obtained from the Cyagen Biosciences (Guangzhou, CN). The above cell lines were cultured in Dulbecco's modified Eagle's medium (DMEM) supplemented with 10% fetal bovine serum (FBS) and 100U/ml penicillin/streptomycin solution (GIBCO, Gaithersburg, US) at 37˚C in a humidified incubator with 5% CO_2_.

### Human specimen collection

We collected 3 pairs of human OS tissue and corresponding paracancerous tissue samples from 3 confirmed OS patients at The First Affiliated Hospital of Guangxi Medical University from August 2020 to August 2022. Additionally, we applied to our studies 60 OS tissue sections which were collected from patients at The First Affiliated Hospital of Guangxi Medical University from January 2012 to December 2019.

### Animal preparation

36 nude mice were purchased from Guangxi Medical University Animal Center and bred in a specific pathogen-free (SPF) animal laboratory. Before the animal experiment, we collected nude mice that weighed 15-20 g and both sexes were used for the in vivo experiment.

### Primers, antibodies, lentivirus (LV) and adenovirus (ADV)

The primers, antibodies, LV and ADV used in the present study are listed in Supplementary Table 1.

### Bioinformatic analysis

In this study, the OS-related microarray dataset was obtained from Gene Expression Omnibus (GEO) and employed to perform bioinformatic analysis. The dataset GSE28425 was subjected to perform differential analyses using R package “DESeq2”, with a significant criteria of |log2 (fold change) |> 1 and *P*-value < 0.05, thereby screening the key miRNA. Furthermore, the relevance between the key miRNA expression level and OS prognosis was investigated by survival analysis using R package “survival” based on the dataset GSE39052. Subsequently, the key downstream mRNA was screened and selected based on the MicroRNA Target Prediction Database (miRDB), followed by the clinically pathological analysis by R package “compare group” based on the clinical data matched with 60 OS sections (*P*-value < 0.05). The survival analysis of key mRNA was subsequently conducted based on SPSS (*P*-value < 0.05). The visualization of the above bioinformatic analysis was implemented by R software.

### Extraction of exosome in h143B cell

The h143B cells were seeded in culture dishes supplemented with DMEM at 37˚C with 5% CO_2_. After reaching 70% confluence, the cells were subjected to a medium change and incubated for a duration of 3 days. Low-speed centrifugation and high-speed centrifugation were sequentially carried out to obtain the supernatant. After filtration, the sample was treated with ultracentrifugation for 90 min. We removed the supernatant and resuspended the cells with PBS to perform secondary ultracentrifugation, followed by collecting and resuspending the exosome precipitation.

### Transmission electron microscopy (TEM) observation

The TEM was employed to observe and documented the exosome morphological characteristics and mitochondrial morphological alternation of the h143B cells in the scale bar 200 nm/50 nm and 500 nm, respectively.

### Nanoparticle tracking analysis (NTA)

NTA was conducted to determine the hydrodynamic size and particle concentration of exosomes using the ZetaView nanoparticle tracking analyzer (Particle Metrix, Meerbusch, GER). Briefly, the analyzer was calibrated with the diluted standard solution after which we diluted the exosome sample to make the number of particles displayed in the instrument interface between 50–400. Subsequently, the analyzer automatically completed the test process, analyzed the data, and then produced the test report.

### Co-culture assay

The co-culture assay was performed to determine whether extracted exosomes can enter OS cells. After revival and passaging, the h143B cells were seeded in a culture dish supplemented with DMEM and then cultured to complete adherence. Meanwhile, the exosome extracts labeled with red PKH26 stain (MKCJ8712, Sigma, St. Louis, Missouri, USA) were added for co-culture with h143B cells. After incubation for 24 h, the culture was washed with PBS 3 times, and was subsequently observed and documented with micrographs using fluorescence microscopy.

### Exosome microarray

To identify the key regulatory molecule in exosomes, human OS tissue, and corresponding paracancerous tissue samples were used as the material of the exosome microarray to determine the aberrant expression of the upstream miRNA in the exosome. Briefly, we prepared the tissue sample as cell suspension followed by extraction of exosomes using the method as described above. We then extracted total RNA extraction and performed RNA sequencing through Agilent miRNA expression profiling microarray (Agilent Technology, Inc., Palo Alto, USA). The raw data was collected using Agilent Feature Extraction software.

### Dual luciferase assay

According to the protocol of Dual-Luciferase Reporter Assay Kit (Hanheng Biotechnology, Shanghai, CN) provided by the manufacturers, we mix the 10uL h-key mRNA-3UTR plasmid with 5 pmol key miRNA/Negative Control (NC) and specific transfection reagent. The above mixture was added into the DMEM medium of 293 T cells, which was subsequently incubated at 37 ˚C with 5% CO_2_. After 48 h, we collected and lysed the cells, mixed the lysate with Lysis Buffer, and centrifuged it at 12,000 rpm. We then added Luciferase Reaction Reagent/Luciferase Reaction Reagent II, and measured the signal intensity of fLuc and rLuc by a microplate reader.

### Western blot (WB)

The exosome, OS cells, osteoblast, OS tissue and corresponding paracancerous tissue were separately prepared for the lysate. After centrifugation, the supernatant was used to perform electrophoresis with 10% or 15% sodium dodecyl sulfate (SDS)-polyacrylamide gels. After that, the proteins were transferred to the polyvinylidene fluoride (PVDF) membrane which was subsequently treated with corresponding primary antibodies and secondary antibodies. Ultimately, the membranes were rinsed with tris buffered saline with tween (TBST) and immersed in enhanced chemiluminescence (WBKLS0500; Pierce, Rockford, IL, USA). The images were obtained with the Bio-Rad ChemiDoc imaging system (Bio-Rad, Hercules, US) and the result was further analyzed by the Image-J software.

### Transcription-quantitative polymerase chain reaction (RT-qPCR)

We respectively processed the OS cells, osteoblast, OS tissue, and corresponding paracancerous tissue and obtained lysate, which was subsequently used to extract total RNA using RNAeasy Animal RNA Isolation Kit with Spin Column(Beyotime, Shanghai, CN). The extracted mRNA was reversely transcribed into cDNA by applying the kit HiScript® III RT SuperMix for qPCR (+ gDNA wiper). Start-up reagent: ChamQ Universal SYBR qPCR Master Mix (Vazyme Biotech, Nanjing, CN). Whilst, the miRNA was reversely transcribed into complementary DNA (cDNA) using the miRNA First Strand cDNA Synthesis kit (Tailing Reaction)(Sangon biotech, Shanghai, CN). We then utilized the 7300Plus Real-Time PCR System (Thermo Fisher Scientific, Shanghai, CN) to carry out quantitative Polymerase Chain Reaction (qPCR).

### Immunohistochemistry (IHC)

The OS tissue and corresponding paracancerous tissue derived from human and nude mice models were used as material for IHC. We started the embedding machine to melt the paraffin which was used to immerse the fixed and dehydrated tissue. After wax immersion and embedding, the tissue was used to prepare sections with a microtome. Subsequently, the sections were dewaxed, rehydrated, and placed in a pot with repair solution and heated to 96˚C. After cooling down, sections were undergone inactivation of endogenous enzymes and were blocked using a 10% goat serum blocking solution. We then added the primary antibody and incubated them at 4˚C overnight followed by the addition of the secondary antibody. All sections were treated with 3,3’-diaminobenzidine (DAB) solution and hematoxylin to stain. After dehydration and sealing by gum, the sections were examined and photographed under microscopy.

### LV and ADV transfection

The LV and ADV for miR-144-3p/ZEB1 knockdown or overexpression were purchased from Hanheng Biotechnology (Shanghai, CN). The h143B cells were transfected with LV or ADV to obtain cells harboring different levels of miR-144-3p or ZEB1, followed by the selection of stable expression cells according to the manufacturer’s instructions.

### Cell counting kit-8 (CCK-8) assay

The Cell Counting Kit-8 (Uelandy, Suzhou, CN) was used to measure the viability of transfected cells. Here, the cell suspension was seeded into a 96-well flat-bottomed plate. After incubating overnight at 37˚C with 5% CO_2_, 10 μL CCK-8 solution was added per plate, and cells were incubated again for 3 h. We then measured the absorbance at 450 nm using a microplate reader at 0 h, 24 h, 48 h, 72 h, and 96 h.

### Transwell assay

The cell imigration and invasion assay was performed with Corning Matrigel Basement Membrane Matrix (Corning Inc, Corning, NY, USA). The Matrigel was thawed, diluted, and added to the growth surface of the transwell chamber (Labselect, Hangzhou, CN) followed by resting at 37˚C for 1 h (skip above protocol in cell migration assay). The h143B cells in the logarithmic growth phase were collected to perform trypsin digestion and serum-free medium resuspension. After that, we added 100 μL cell suspension to the upper chamber and added 600 μL 20% serum medium in the lower chamber. After 24 h incubation, we removed the culture and washed the transwell chamber with PBS. The polyethylene terephthalate (PET) membrane was immersed in 70% methanol to fix the cells, which were subsequently stained by crystal violet solution. Ultimately, we used the inverted fluorescence microscope to obtain the final result.

### Glutathione (GSH) / glutathione disulfide (GSSG) assay

The GSH and GSSG assay was performed using Toal glutathione / Oxidized glutathione Assay Kit (Jiancheng Bioengineering Institute, Nanjing, CN). According to the instructions, we prepared the required reagents and GSH/GSSG standard sample. After mixing the cells with homogenization medium and centrifuging, the supernatant was mixed with the reagents and standard sample mentioned above. The resulting mixture was then measured for the delta of absorbance at 30 s and 10 min 30 s using a microplate reader at 412 nm. Subsequently, we calculated the level of GSSG and GSH according to the formula provided by manufacturers, thereby determining the ratio of GSH/GSSG.

### Ferrous iron (Fe^2+^) level assay

The Cell Total Iron Colorimetric Assay Kit was purchased from Elabscience (Wuhan, CN) to conduct Fe^2+^ level assay. The transfected cells were lysed on ice and centrifuged at 15000 g. We then collected the supernatant and added it to a reagent containing iron reductase. After incubating for 40 min, the optical density was measured by a microplate reader at 593 nm.

### Malondialdehyde (MDA) assay

We applied the Malondialdehyde Assay Kit (Jiancheng Bioengineering Institute, Nanjing, CN) to determine the level of MDA, a quantitative biomarker of ferroptosis mechanism. According to the manufacturer's protocol, we prepared the mixing solution with transfected cells and a specific reagent provided by the kit. We then performed a water bath, cooled the sample, and centrifuged it at 3500 rpm. After centrifugation, we measured the absorbance of the sample at 532 nm using a microplate reader.

### Reactive oxygen species (ROS) assay

The ROS assay was carried out using Reactive Oxygen Species Assay Kit C1300 (Beijing Solarbio Science and Technology, Beijing, CN). We initially harvested cells to prepare the cell suspension. After centrifugation and precipitation, the cells were collected, and we added the Dihydroethidium (DHE) probes reagent followed by incubation for 30 min. We then carried out a secondary centrifugation and collected the precipitation, which was further made into the cell suspension. Ultimately, the inverted fluorescence microscope was used to observe and obtain fluorescence micrographs (scale bars 100 μm and 50 μm).

### Construction of the cell-line-derived tumor xenograft (CDTX) model

The OS model of nude mice was constructed using the CDTX model. After transfection, the h143B cells were prepared into a single-cell suspension and subcutaneously injected into the left proximal tibia of 36 (3 mice per group) 4-weeks-old nude mice (1 × 10^7^ cells per mouse).

### Magnetic resonance imaging (MRI)

After the construction of murine OS models, the nude mice were treated with isoflurane and then underwent MRI. The RadiAnt software was used to obtain the image of the T2 longitudinal axis and the software 3Dslicer was used to calculate the tumor volume. Following ethical regulations, the maximum diameter of each nude mouse’s tumor should not exceed 20 mm.

### Statistical analysis

The numeric results in present study are expressed as mean ± standard deviation (SD). We used a two-tailed Student's t-test to analyze statistical comparisons between groups (*P*-value < 0.05). The relevance between miRNA expression and mRNA level was quantified by Spearman's correlation coefficient using R software.

## Results

### Identification and validation of miR-144-3p as the critical regulatory factor in OS

**Fig. 2:**
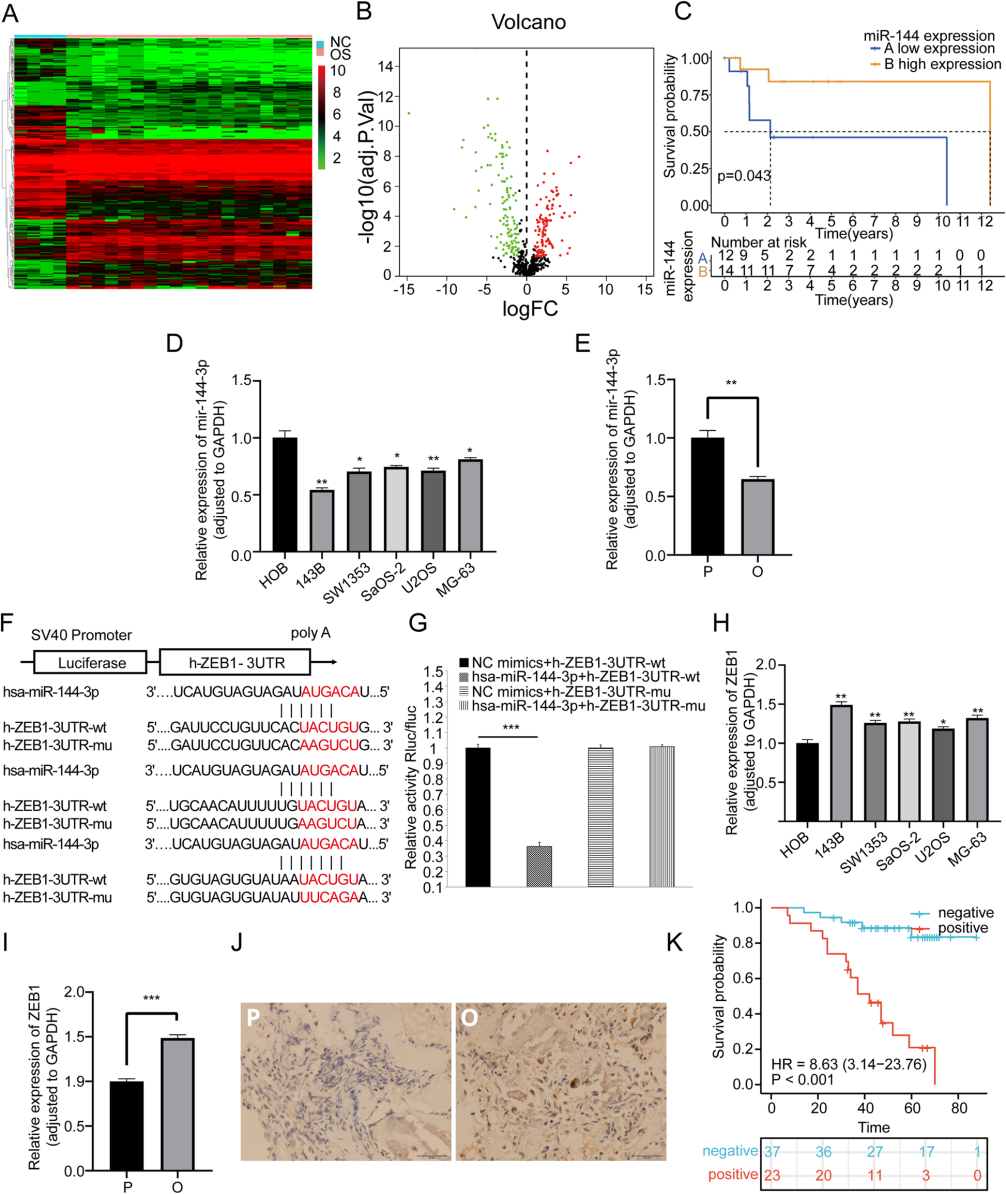
Identification and validation of key miRNA and downstream mRNA. **A** The heatmap and **B** volcano plot showing the profile of differentially expressed genes based on dataset GSE28425 in the context of OS. **C** The Kaplan–Meier plot related miR-144-3p expression based on dataset GSE39052. The RT-qPCR results of key miRNA based on **D** five OS cell lines and one osteoblastic cell line, as well as **E** human OS tissue and paracancerous tissue samples. **F** The binding site sequence of miR-144-3p and ZEB1. **G** The dual luciferase assay indicated that miR-144-3p can bind to ZEB1. The RT-qPCR results of key mRNA based on **H** five OS cell lines and one osteoblastic cell line, as well as **I** human OS tissue and paracancerous tissue samples. **J** IHC micrographs based on 60 tissue sections. **K** The Kaplan–Meier plot for ZEB1 based on the clinical data of 60 patients corresponding to tissue sections

The differential analysis was employed based on bioinformatic technology (Fig. 2A, B) and the result identified the aberrantly low expression of miR-144-3p in OS. We then perform a survival analysis based on miR-144-3p expression level (Fig. 2C), the patients with low-level miR-144-3p were closely associated with a dismal survival time in comparison to patients with high-level miR-144-3p, which suggested that miR-144-3p is a valuable prognostic biomarker for OS. Moreover, the subsequent RT-qPCR assay for 5 OS cell lines and osteoblast, as well as for OS tissue and matched paracancerous tissue also verified the downregulation of miR-144-3p in OS, indicating that miR-144-3p can be considered the critical regulator in OS (Fig. 2D, E).

### Identification and validation of ZEB1 as downstream target of miR-144-3p

To determine the target of miR-144-3p, the miRDB database was applied to predict downstream mRNA based on the rank of target score, and the ZEB1 was selected as the key mRNA among candidate mRNAs. Further dual luciferase assay exhibited the binding site (Fig. 2F) and showed the result that miR-144-3p significantly decreased the expression level of luciferase in h-ZEB1-3UTR-WT (*P*-value < 0.001) relative to NC groups, suggesting that there was a binding effect between them (Fig. 2G). The RT-qPCR for 5 OS cell lines and osteoblast, as well as for OS tissue and corresponding paracancerous tissue, showed aberrantly high expression of ZEB1 in OS (Fig. 2H, I). Meanwhile, the result of IHC was consistent with RT-qPCR (Fig. 2J) and the survival analysis based on 60 OS tissue sections connected the high-level expression of ZEB1 with worse prognosis (Fig. 2K, Tables 1 and 2).

**Table 1:** Associations between clinically pathological characteristics and ZEB1 expression

Variable	Number of patients	ZEB1 expression		*P* value
		Positive	Negative	
	*N* = 60	*n* = 23	*n* = 37	
**Age**				0.288
> 16 years	30 (50.0%)	14 (60.9%)	16 (43.2%)	
≤ 16 years	30 (50.0%)	9 (39.1%)	21 (56.8%)	
**Gender**				0.097
Female	25 (41.7%)	6 (26.1%)	19 (51.4%)	
Male	35 (58.3%)	17 (73.9%)	18 (48.6%)	
**Relapse**				0.004
No	45 (75.0%)	12 (52.2%)	33 (89.2%)	
Yes	15 (25.0%)	11 (47.8%)	4 (10.8%)	
**Metastasis**				0.013
No	39 (65.0%)	10 (43.5%)	29 (78.4%)	
Yes	21 (35.0%)	13 (56.5%)	8 (21.6%)	
**TNM**				0.025
I	22 (36.7%)	13 (56.5%)	9 (24.3%)	
II/III	38 (63.3%)	10 (43.5%)	28 (75.7%)	
**Site**				0.435
Else	9 (15.0%)	5 (21.7%)	4 (10.8%)	
Femur/Tibia	51 (85.0%)	18 (78.3%)	33 (89.2%)	
**Size**				0.288
> 6 cm	30 (50.0%)	14 (60.9%)	16 (43.2%)	
≤ 6 cm	30 (50.0%)	9 (39.1%)	21 (56.8%)

**Table 2:** Univariable and multivariable Cox regression analysis of clinical characteristics and ZEB1 expression

Variable	Univariate analysis			Multivariate analysis		
	HR	95% CI	*P* value	HR	95% CI	*P* value
**ZEB1**						
Positive *vs* Negative	8.63	3.14–23.76	0.00003	5.82	1.82–18.58	0.003
**Age**						
> 16 years *vs* ≤ 16 years	1.23	0.53–2.84	0.62871			
**Gender**						
Female *vs* Male	0.84	0.36–1.96	0.67883			
**Relapse**						
Yes *vs* No	4.97	2.13–11.61	0.00021	0.94	0.30–2.95	0.911
**Metastasis**						
Yes *vs* No	8.08	3.12–20.93	0.00002	12.05	1.28-113.05	0.029
**TNM stage**						
I *vs* II/III	0.19	0.08–0.47	0.00034	2.14	0.30-15.47	0.045
**Location**						
Femur/Tibia *vs* Else	0.47	0.17–1.29	0.14147	0.93	0.30-2.86	0.895
**Tumor size**						
> 6 cm *vs* ≤ 6 cm	0.53	0.22–1.26	0.14862	0.84	0.32-2.21	0.722

### Extraction and validation of miR-144-3p in OS-mediated exosome

**Fig. 3:**
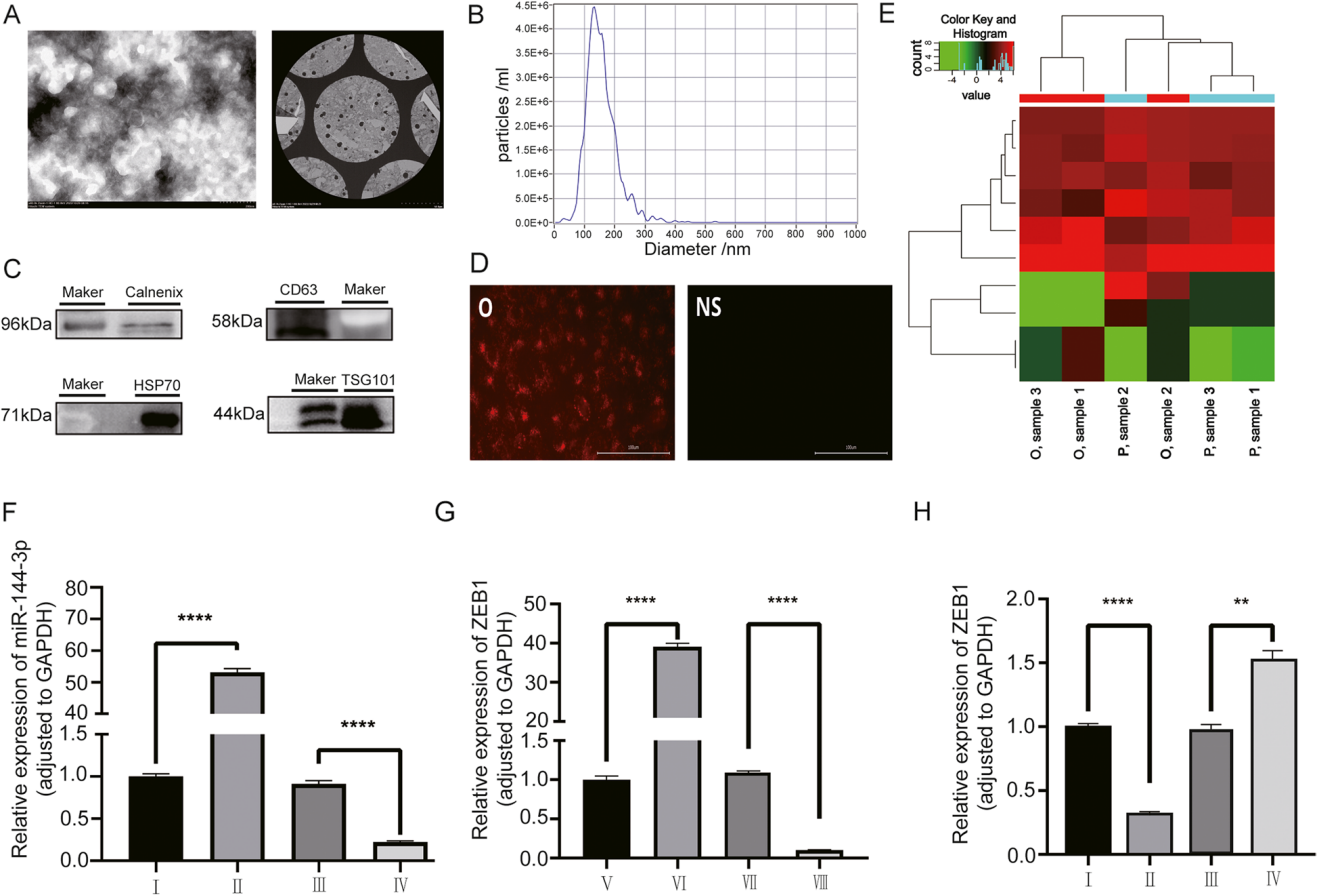
The validation of exosome expression, function and downstream miR-144-3p/ZEB1 axis. **A** TEM micrographs of exosomes. **B** The NTA indicated the particle size, volume, and concentration of exosomes. **C** The WB of exosomes characteristic proteins. **D** The co-culture assay indicated that labeled exosomes can enter OS cells.** E** The heatmap of exosome microarray. The RT-qPCR results for validation of transfection **F** miR-144-3p in Group I-IV and **G** ZEB1 in Group V-VIII. The RT-qPCR results for validation of downstream **H** ZEB1 in Groups I-IV

To detect the expression of exosomes released from OS, the exosomes were isolated from h143B cells. Through TEM, the vesicle morphological feature was observed and described as a “bilayer saucer shape” under TEM in a scale bar of 200 nm and 50 nm (Fig. 3A), which was consistent with typical exosome presentation. The NTA reported that the median particle size of extracted exosomes was determined to be 131.8 nm, and the concentration of exosomes was 4.5E^+6^ particles/mL (Fig. 3B). The WB assay showed that the exosomal characteristic proteins (CD63, HSP70 and TSG101) were highly expressed in the exosome lysate while the cytoplasm characteristic protein calnexin exhibit low expression level (Fig. 3C). In subsequent co-culture assay, significant red fluorescent staining could be observed in OS cells, which validated that exosome can act as a carrier to transport biological molecules into cells (Fig. 3D). To validate the aberrant expression of miR-144-3p in the exosome, we conduct an exosomal microarray (Fig. 3E) and the result showed that the expression of miR-144-3p was significantly decreased in exosome derived from human OS tissue compared with paracancerous tissue (*P*-value < 0.05).

### Transfection and verification of miR-144-3p/ZEB1 axis

By transfection with LV or ADV, we knocked down or overexpressed the expression of miR-144-3p or ZEB1 in distinct groups of h143B cells and the significant transfection effectiveness was verified by RT-qPCR (Fig. 3F, G). Furthermore, to validate the regulatory effect of miR-144-3p on ZEB1, the RT-qPCR was applied to detect the ZEB1 expression level in the miR-144-3p overexpression group, miR-144-3p knockdown group and corresponding NC groups. The result showed that the expression of ZEB1 was reversely correlated with miR-144-3p (Fig. 3H), which demonstrated the regulatory relationship between miR-144-3p and ZEB1.

### MiR-144-3p inhibits OS proliferation, invasion and migration

**Fig. 4:**
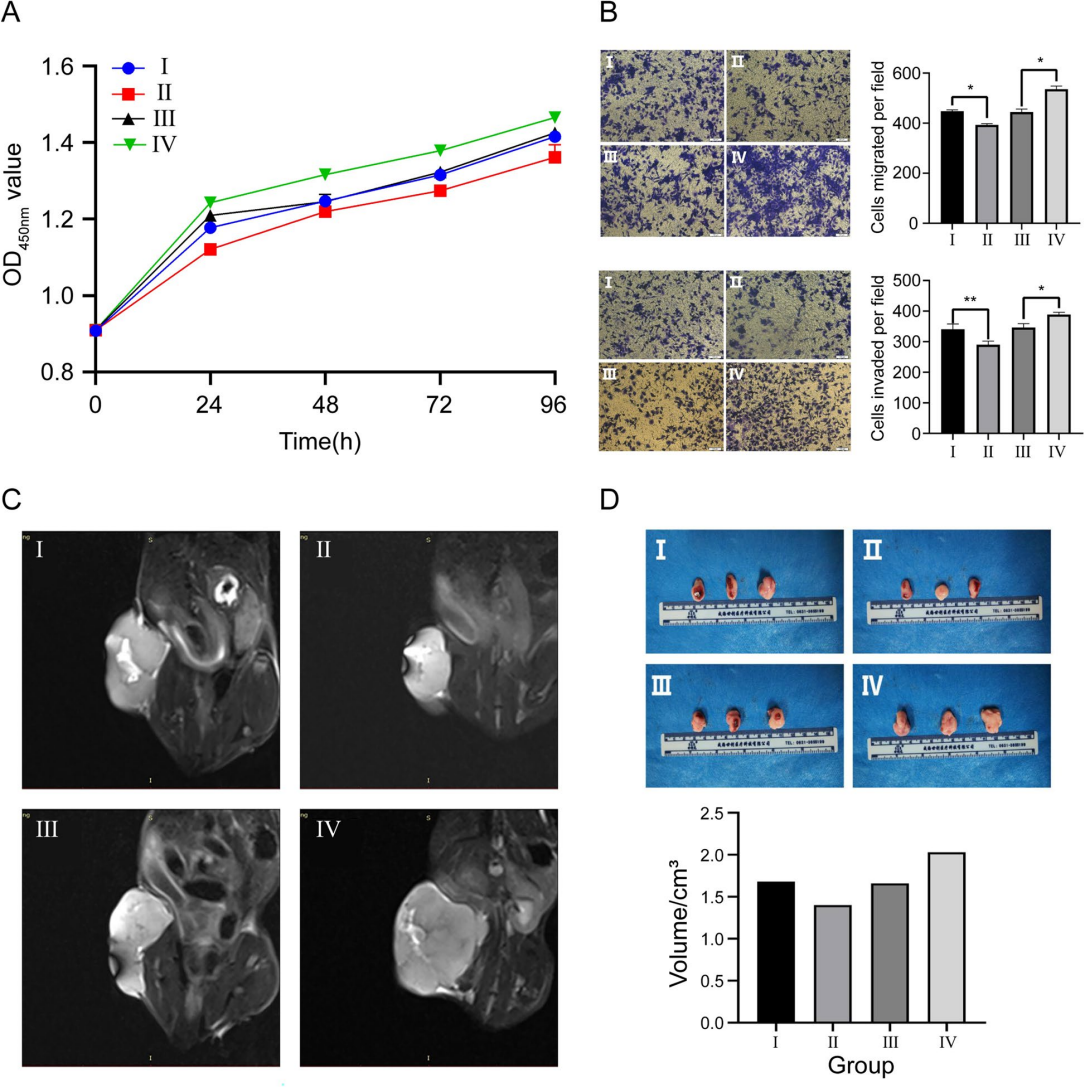
Effect of miR-144-3p expression on OS proliferation, invasion and migration. **A** CCK-8 assay. **B** Transwell assay of migration and invasion. **C** MRI images. **D** Gross samples

The CCK-8 assay was performed to detect the viability of OS cells in vitro. We found that the overexpression of miR-144-3p can lead to the decreased level of OS viability (Fig. 4A). Subsequently, we conducted a transwell assay to explore the effect of miR-144-3p on OS cell migration and invasion in vitro (Fig. 4B), and the result indicated that higher levels of miR-144-3p in h143B cells were associated with lower migration and invasion tendency. To further elaborate the anti-tumor function of miR-144-3p in vivo, the CDTX model was constructed based on nude mice. We carried out MRI to evaluate the volume of OS in distinct transfection groups and found that the OS derived from miR-144-3p overexpression group exhibited a smaller volume than the OS derived from the corresponding NC group (Fig. 4C), which was similarly consistent with the tumor results from gross anatomy (Fig. 4D). In all of the assays mentioned above, the miR-144-3p knockdown group exhibited an opposite tendency to the miR-144-3p overexpression group, suggesting that miR-144-3p suppresses the proliferation, invasion, and migration of OS cells.

### MiR-144-3p positively regulates intracellular ferroptosis mechanism

**Fig. 5:**
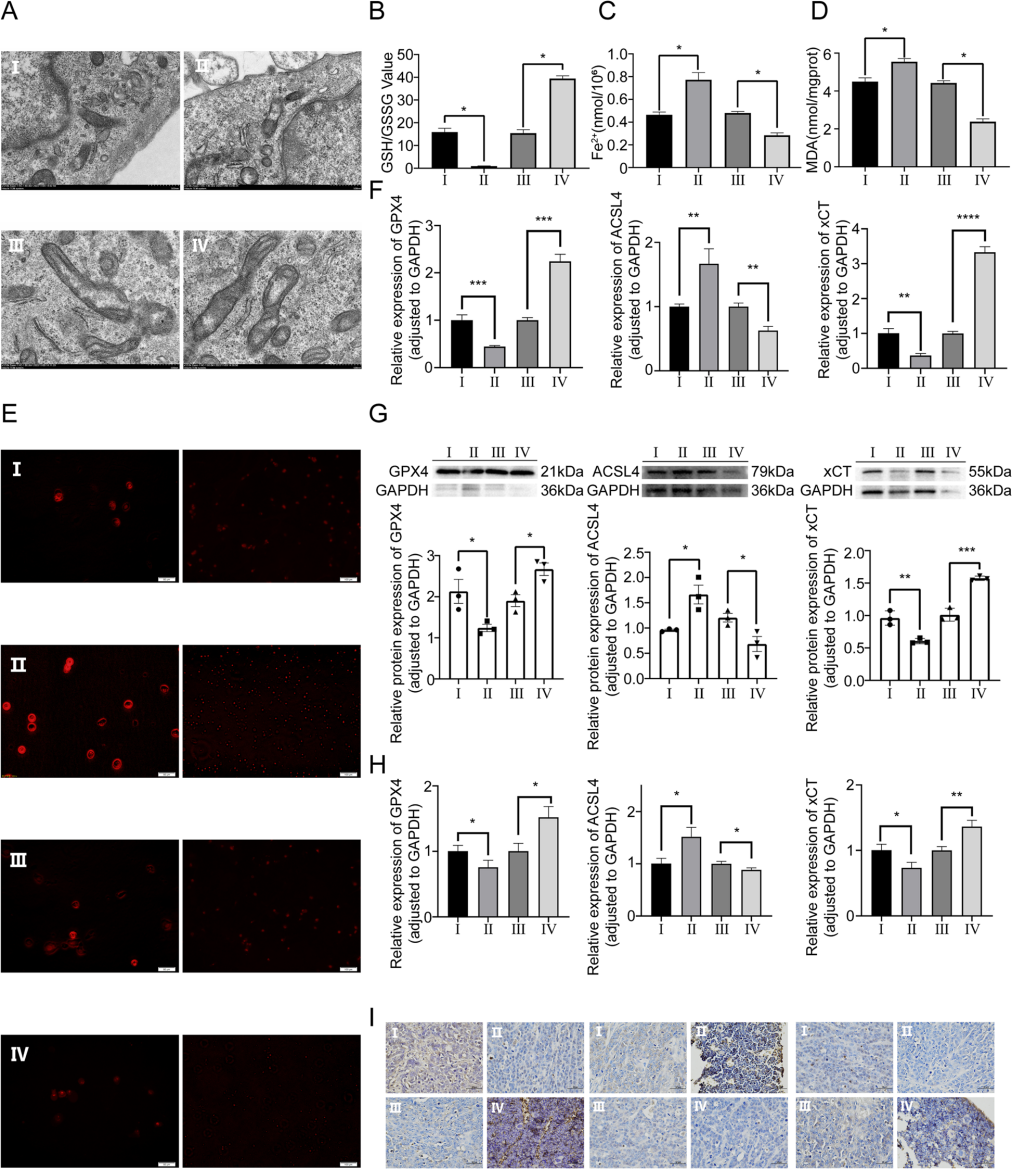
Effect of miR-144-3p expression on ferroptosis mechanism. **A** TEM micrographs of mitochondria. **B** Glutathione assay. **C** Fe^2+^ level assay. **D** MDA assay. **E** TEM micrographs of immunofluorescence for ROS. **F** The RT-qPCR and **G** WB assay of GPX4, ACSL4, and xCT in vitro experiment. **H** The RT-qPCR and **I** IHC assay of GPX4, ACSL4, and xCT in vivo experiment

To investigate whether miR-144-3p inhibits OS growth via modulating ferroptosis mechanism, the TEM was applied to observe intracellular morphological alteration (Fig. 5A). The mitochondrial morphology in OS cells with a higher level of miR-144-3p manifested as more significant ferroptosis-related aberrance compared with other transfection groups, including mitochondria atrophy, mitochondria cristae reduction, and mitochondrial outer membrane rupture. By GSH assay, we observed a lower GSH/GSSG ratio in OS cells with overexpression miR-144-3p, compared with corresponding NC groups (Fig. 5B). Furthermore, the Fe^2+^ level assay was performed to identify the association between iron metastasis homeostasis and expression of miR-144-3p, and the result showed that the miR-144-3p can positively regulate the Fe^2+^ level in OS cells (Fig. 5C). In subsequent MDA assay, the relative MDA level was more prominent in OS cells with the increased level of miR-144-3p relative to the NC groups (Fig. 5D). The ROS assay was conducted to determine the peroxidative stress within OS cells, the miR-144-3p can significantly elevate the intracellular ROS level (Fig. 5E). Ultimately, the findings from the in vitro experiment demonstrated that the levels of ferroptosis-related molecules (GPX4, ACSL4 and xCT) in transfected OS cells were evaluated using RT-qPCR and WB assay. The results revealed a positive correlation between the expression of miR-144-3p and the pro-ferroptosis molecule ACSL4, while a negative correlation between anti-ferroptosis molecules (xCT and GPX4) and the expression level of miR-144-3p (Fig. 5F, G). Meanwhile, in vivo experiment, the tissue collected from the CDTX model was used to perform RT-qPCR and IHC to determine ferroptosis-related molecules (GPX4, ACSL4 and xCT) as well, and the result was consistent with in vitro experiment (Fig. 5H, I). The assays described above showed that the group with miR-144-3p knockdown and its corresponding NC exhibited a trend that was opposite to that of the group with miR-144-3p overexpression and its NC, which sufficiently demonstrated that miR-144-3p facilitates ferroptosis by integration of multiple effects, including metastatic regulation of GSH/GSSG, Fe^2+^ and ROS levels.

### ZEB1 positively regulated OS proliferation, migration and invasion in vitro and in vivo

**Fig. 6:**
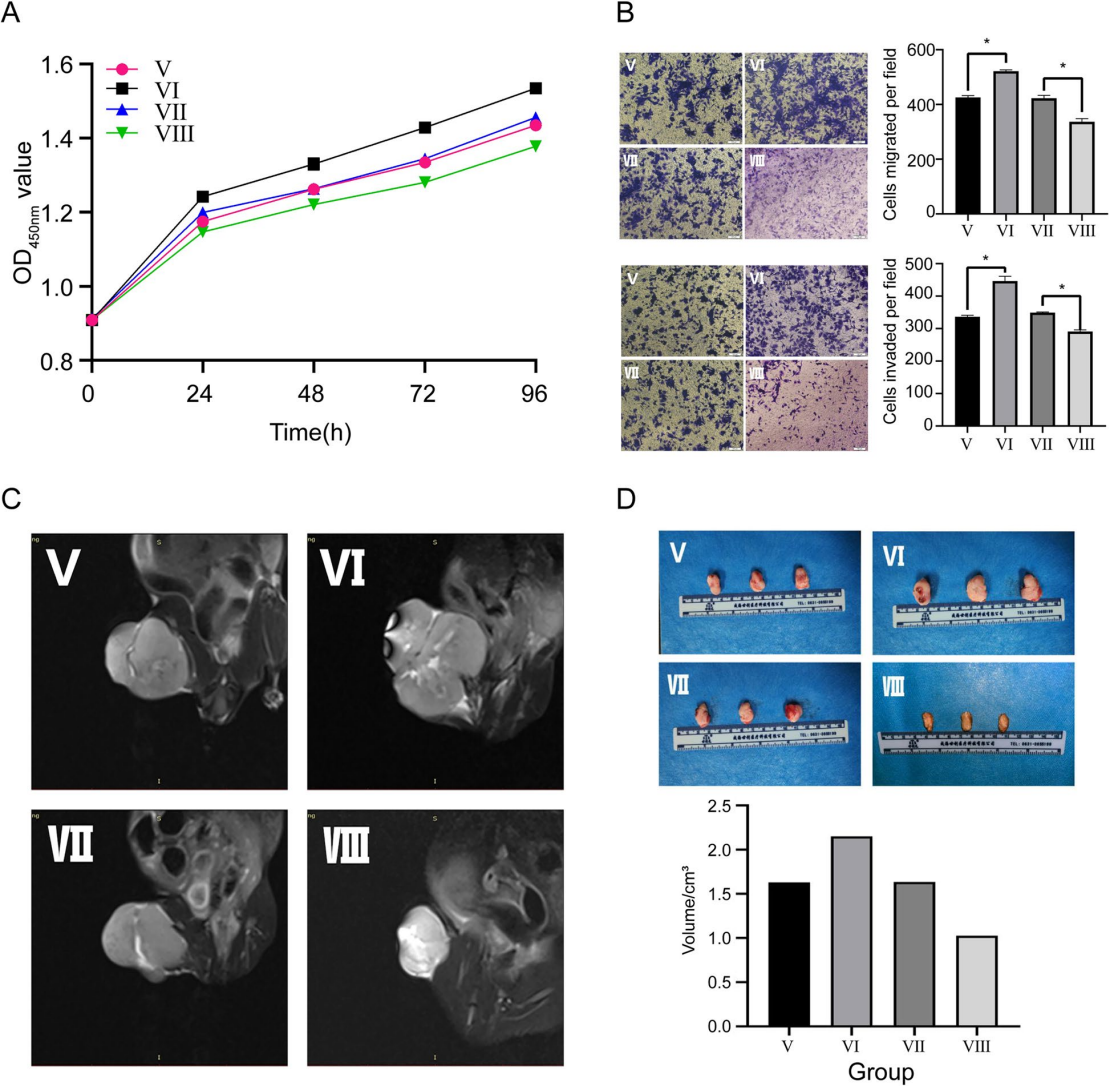
Effect of ZEB1 expression on OS proliferation, invasion and migration. **A** CCK-8 assay. **B** Transwell assay of migration and invasion. **C** MRI images. **D** Gross samples

The CCK-8 assay and transwell assay was performed to identify the viability, migration and invasion of h143B cells in distinct transfection grouping. We found that the viability, migration and invasion of OS cells in the ZEB1 overexpression group was more prominent than that in the NC group (Fig. 6A, B), indicating that ZEB1 can positively regulate the pro-oncogenic phenotype of OS cells. Subsequently, we carried out the in vivo experiment to validate the positive regulatory effect of ZEB1 on OS growth and development. By measuring the volume based on MRI and tumor size based on the gross sample, it can be observed that the OS derived from h143B cells highly expressing ZEB1 exhibits a more prominent volume in the MRI image and larger tumor size in the gross sample, compared with matched NC group (Fig. 6C, D). Meanwhile, in the aforementioned assays, the ZEB1 knockdown group and corresponding NC group exhibited the opposite result to the ZEB1 overexpression group and NC group. Thus, we concluded that ZEB1 can positively regulate OS progression in vitro and in vivo.

### The expression of ZEB1 was reversely correlated with intracellular ferroptosis level

**Fig. 7:**
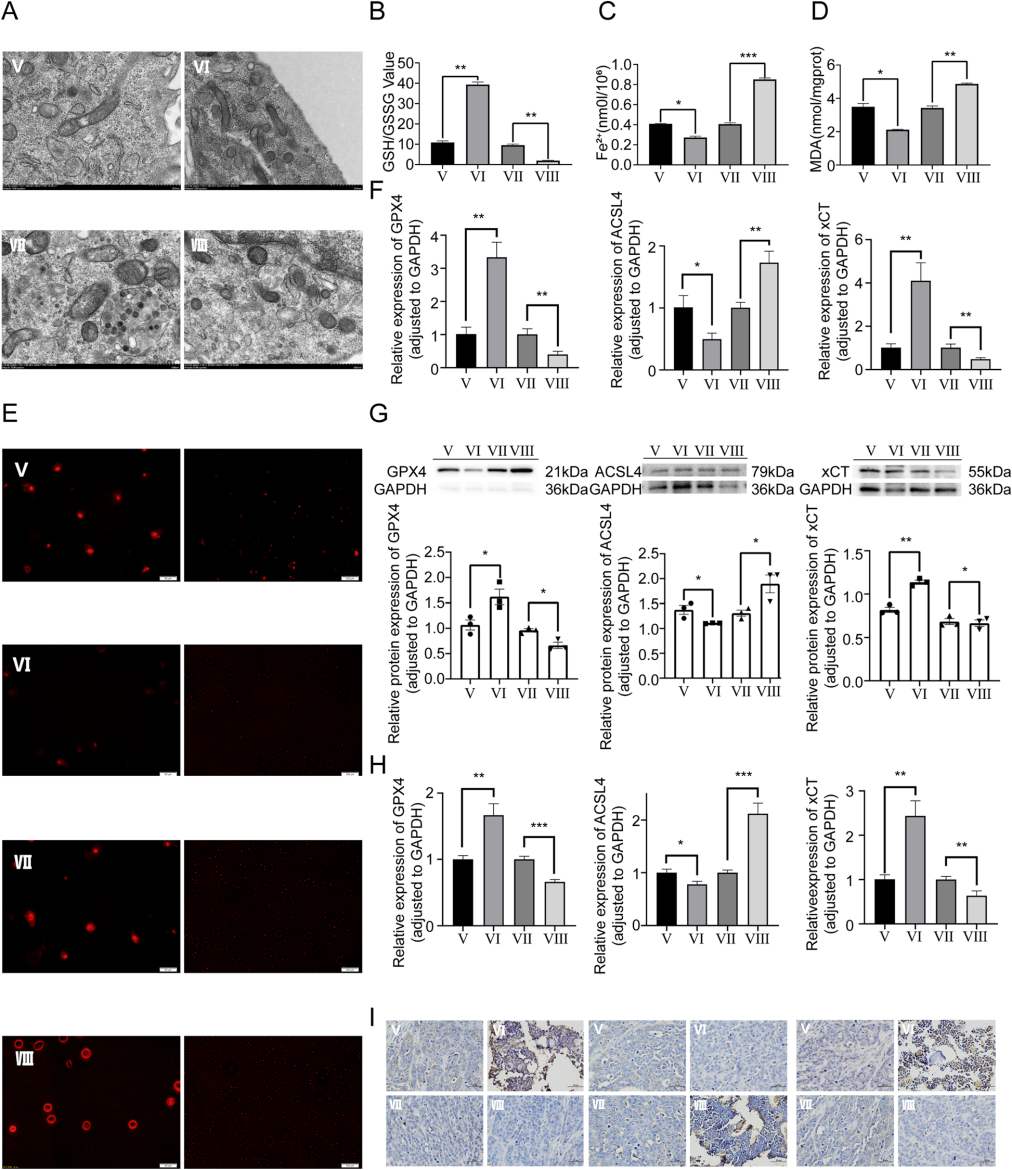
Effect of ZEB1 expression on ferroptosis mechanism. **A** TEM micrographs of mitochondria. **B** Glutathione assay. **C** Fe^2+^ level assay. **D** MDA assay. **E** TEM micrographs of immunofluorescence for ROS. **F** The RT-qPCR and **G** WB assay of GPX4, ACSL4, and xCT in vitro experiment. **H** The RT-qPCR and (I) IHC assay of GPX4, ACSL4, and xCT in vivo experiment

To explore the role of ZEB1 in the execution of ferroptosis, we observed more substantial ferroptosis-specific morphological alteration of mitochondria in the ZEB1 knockdown group relative to other transfection groups through TEM (Fig. 7A). In the GSH assay, the result indicated that the GSH/GSSG ratio was positively correlated with the expression level of ZEB1 (Fig. 7B). Meanwhile, in the Fe^2+^ level assay, the MDA assay and the ROS assay, the intracellular levels of these ferroptosis-relative indicators were significantly decreased in the ZEB1 overexpression group compared to the NC group, indicating that ZEB1 inhibits the ferroptosis by affecting glutamine metabolism and Fe^2+^, MDA and ROS level (Fig. 7C-E). Subsequently, based on OS cells in diverse transfection groups, the RT-qPCR and WB assay specific for ferroptosis-related molecules (GPX4, ACSL4 and xCT) was performed and the result showed that the overexpression of ZEB1 caused the lower expression level of ACSL4 and higher expression level of xCT and GPX4 (Fig. 7F-G). Furthermore, the RT-qPCR and IHC assay based on the CDTX model in vivo experiment was consistent with the above in vitro experiment (Fig. 7H-I). It is noteworthy that the above assays showed that the group with ZEB1 knockdown and its corresponding NC exhibited a trend that was opposite to that of the group with ZEB1 overexpression and its NC, demonstrating that ZEB1 inhibits ferroptosis by regulating glutamine metabolism, Fe^2+^ homeostasis, MDA level and ROS level.

### ZEB1 rescues the effect of miR-144-3p on OS via regulating ferroptosis

**Fig. 8:**
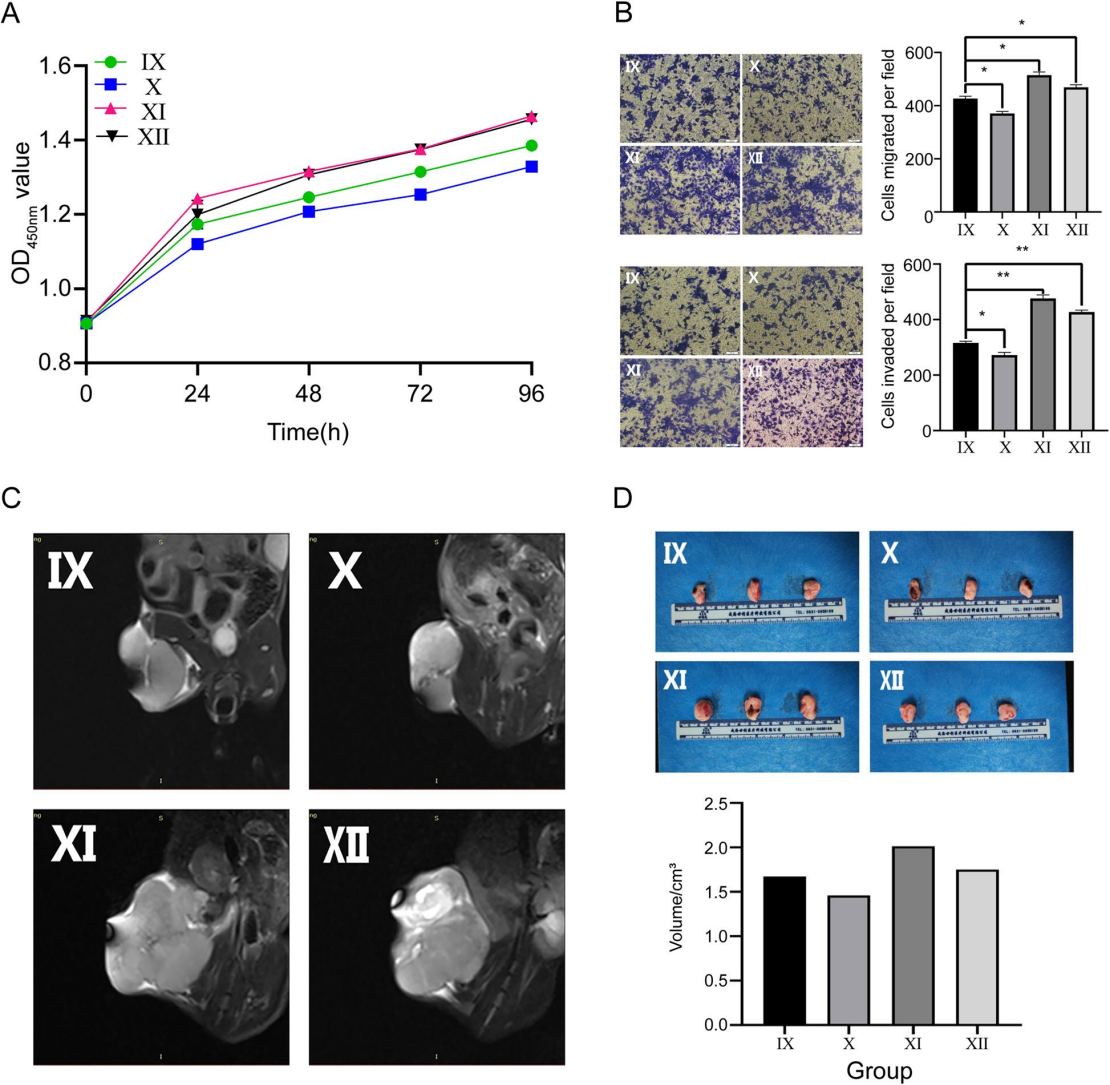
Effect of miR-144-3p/ZEB1 axis on OS proliferation, invasion and migration. **A** CCK-8 assay. **B** Transwell assay of migration and invasion. **C** MRI images. **D** Gross samples

**Fig. 9:**
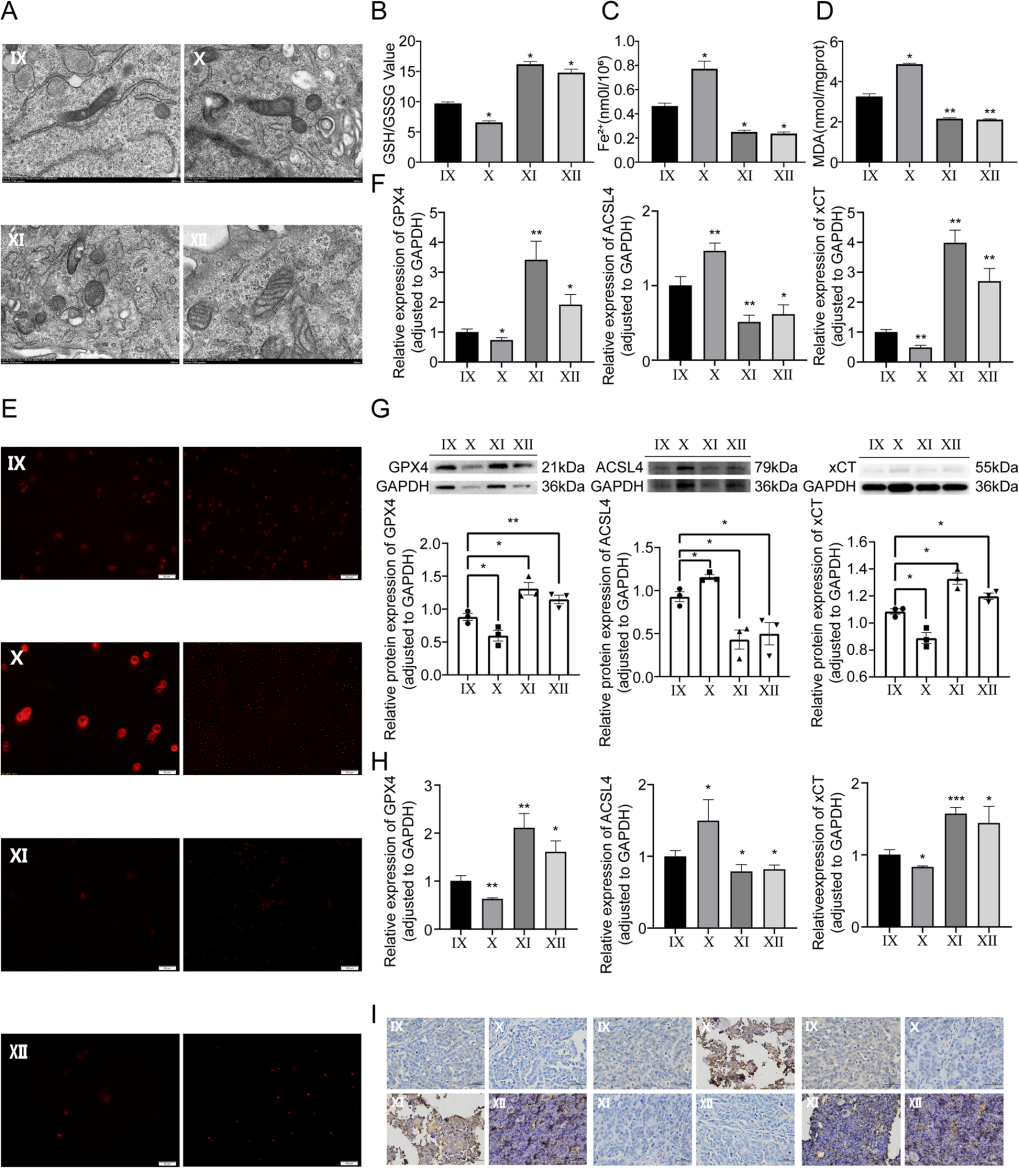
Effect of miR-144-3p/ZEB1 axis on ferroptosis mechanism. **A** TEM micrographs of mitochondria. **B** Glutathione assay. **C** Fe^2+^ level assay. **D** MDA assay. **E** TEM micrographs of immunofluorescence for ROS. **F** The RT-qPCR and **G** WB assay of GPX4, ACSL4, and xCT in vitro experiment. **H** The RT-qPCR and **I** IHC assay of GPX4, ACSL4, and xCT in vivo experiment

To verify the upstream and downstream relationship of the miR144-3p/ZEB1 axis, we designed a functional rescue experiment based on transfection grouping to validate the function of the miR-144-3p/ZEB1 axis. The CCK-8 assay and transwell assay was conducted and the result of which indicated that the overexpression of ZEB1 can rescue the tumor inhibiting effect of miR-144-3p, thereby promoting the proliferation, migration and invasion tendency of OS cells (Fig. 8A, B). The subsequent in vivo experiment demonstrated that the increased expression of miR-144-3p can inhibit OS growth via regulating ZEB1 (Fig. 8C, D). To further confirm that the mechanism of OS development is regulated by promoting ferroptosis mediated by the miR-144-3p/ZEB1 axis, we applied the TEM to observe mitochondrial morphology and determine intracellular GSH/GSSG ratio, Fe^2+^ level, MDA level and ROS level. The result indicated that the ZEB1 can restore the effect of miR-144-3p on the ferroptosis-related pathway (Fig. 9A-E). Ultimately, the OS cells and CDTX tissue of distinct transfection groups were used as the material of RT-qPCR, WB assay and IHC assay to detect ferroptosis-related molecules (GPX4, ACSL4 and xCT) in vitro experiment and in vivo experiment (Fig. 9F-I). The result further demonstrated that ZEB1 can rescue the effect of miR-144-3p on OS via regulating ferroptosis.

## Discussion

Cumulative studies have found that miRNA expression levels are closely linked to the formation and progression of tumors as well as the deterioration of human malignant tumors. The expression of serum miRNAs as a diagnostic and prognostic marker of tumors has become a hot topic of research. MiR-144 has been involved in the etiopathogenesis of colorectal cancer, breast cancer, and pancreatic cancer, and its expression in these cancers is often significantly reduced [24, 25]. In OS, research has shown that miR-144 can play a role as an inhibitor of tumor proliferation and affect the prognosis of OS patients [13]. In addition, miR-144 can also target mTOR and inhibit the PI3K/protein kinase B (PKB) signaling pathway, thereby suppressing the multiplication of OS cells and promoting their apoptosis [26]. In this study, we found that miR-144-3p had significant differences in its expression in tissues through bioinformatics analysis, and this may be closely related to the modulation of ferroptosis mechanisms in OS. We detected miR-144-3p in OS tissues by using RT-qPCR, WB, and IHC, finding that miR-144-3p was downregulated in OS tissues. In addition, the relative retrospective prognostic data of OS patients suggested that a high level of miR-144-3p was linked to a good prognosis. The above evidence indicates that miR-144-3p may be used as a tumor inhibitor in OS cells.miRNAs mainly play a role in post-transcriptional execution which can promote abnormal protein expression levels via sequence complementation between miRNAs and corresponding target mRNA. We have screened the downstream target molecule ZEB1 of miR-144-3p based on the miRDB database. By targeting ZEB1, miR-144 can limit the proliferation of lung cancer cells [27], we suspected that miR-144-3p and ZEB1 may have a direct regulatory relationship and exert effectiveness together on OS, hence the experimental verification. Through a double luciferase experiment, we verified that miR-144-3p can bind to ZEB1, which serves as the direct target of miR-144-3p. ZEB1, a zinc finger transcription factor, significantly promotes epithelial-mesenchymal transformation. Research shows that ZEB1 is overexpressed in the tumor microenvironment and participates in the formation and development of tumors by promoting tumor proliferation, invasion, and migration [28]. There are only few studies on ZEB1-related molecular effects in the context of OS. At present, it has been found that the nuclear factor kappa-B(NF-κB)/inducible isoform of nitric oxide synthase (iNOS) pathway promotes caspase 3-mediated apoptosis, which can inhibit ZEB1 transcription, thereby inhibiting the proliferation of MG-63 OS cells [29]. By measuring the expression of miR-144-3p and ZEB1, we found that the expression of miR-144-3p in OS tissue was negatively correlated with ZEB1 expression, and overexpression of miR-144-3p could reduce the expression of ZEB1 in OS cells at mRNA and protein levels. Therefore, we speculated that miR-144-3p may inhibit the progression and metastasis of OS by inhibiting the expression of ZEB1.

Some studies show that exosome-mediated miRNAs can play a role by modulating the expression of target genes [30]. The rapid development of exosome research also provides a new opportunity to investigate new tumor markers. Exosomes are related to inflammation, infection, and other processes, which may promote a proliferative environment and the accumulation of mutations, and eventually facilitate the progression of malignant tumors [31]. Tumor-derived exosomes (TDEs) can transport factors into the peripheral blood to participate in the development of migration and can promote tumor cells to escape from the immune system [31]. Some TDEs rich in miRNAs have been considered promising cancer hallmarks [32]. In OS, it has been shown that linc00852 in exosomes is a key intercellular messenger in OS. It not only acts as a regulator of intercellular communication but also mediates vascular remodeling and thus promotes tumor metastasis [33]. However, little is known about the role of miRNAs in exosomes in OS. Therefore, we have studied the mechanism of the miR-144-3p/ZEB1 axis in OS by extracting exosomes from OS tissues and conducting in vitro and in vivo experiments. We conducted the transwell assay and co-culture assay on the exosomes to observe the activity of OS cells’ migration and invasion, thereby elucidating the exosomes’ function. The results showed that the number of cells in the field of vision under the microscope in the Knockdown miR-144-3p group was the largest, indicating that knockdown of miR-144-3p could enhance the invasion and migration ability of cells and accelerate the development of tumors. Overexpression of miR-144-3p was shown to inhibit the growth of OS in nude mice models. According to the results of in vitro and in vivo experiments, we believe that miR-144-3p mediated by exosomes can inhibit the proliferation, invasion, and migration of OS cells.

Known as a recently discovered cell-programmed death mechanism, ferroptosis has been a research hotspot. Its main mechanisms are lipid metabolism disorder, GSH imbalance, and iron metabolism disorder. GPX4 uses GSH as the substrate to repress toxic peroxides into non-toxic hydroxyl fractions, and protect the stability and normal physiological function of the cell membrane from interference and destruction of peroxides, resulting in inhibiting ferroptosis in cancer cells [34]. The deletion of the GPX4 gene can lead to lipid peroxidation, and then induce the ferroptosis pathway in tumorigenesis [35]. Another way of ferroptosis is the accumulation of iron, which is indispensable in the physiological process, but excessive iron will be harmful to the body. Enhancing mechanism of cellular iron export has been shown to increase cell resistance to ferroptosis [36, 37]. Another important mechanism is the aggregation of ROS. The intracellular aggregation of lipid ROS can damage the cell membrane due to the effect of the lipid peroxidation process, ultimately resulting in cell death. ROS mainly passes the Fenton reaction of ferrous ions and lipid peroxidation. When GPX4 and GSH are depleted, the build-up of ROS with cytotoxicity will occur, thus inducing cellular ferroptosis [38]. ACSL4 is a protein that plays a crucial role in regulating the metabolism of fatty acids and lipids. Exosome-mediated miRNA has been found to regulate ACSL4 expression and thus play a role in ferroptosis. For instance, exosomes derived from mesenchymal stem cells have been found to transfer miR-424 to lung cancer cells, which downregulates ACSL4 expression and inhibits ferroptosis [39]. In addition, The ferroptosis protein xCT has also been proven to be regulated by miRNAs mediated by exosomes and play a role in maintaining redox balance. Therefore, by measuring the levels of proteins such as ACSL4 and xCT, the degree of cell ferroptosis can be understood. The present research explored whether miR-144-3p and ZEB1 affect ferroptosis in OS by measuring the related factors of the above pathways and ferroptosis-related proteins. The results verified that overexpression of miR-144-3p can inhibit ZEB1 expression level, and promote the cellular ferroptosis mechanism, thereby suppressing the proliferation of OS.

It should be noted that differences between the Knockdown miR-144-3p group and the NC group in many ferroptosis-related indicators were not very significant. In response, we speculated that the original expression level of miR-144-3p was considerably low in OS cells. When the expression of miR-144-3p is lower than a specific threshold, its continued reduction will no longer have a significant impact on ferroptosis. It may be a self-protection mechanism for cells to avoid ferroptosis, an area that still needs in-depth exploration.

Our research has proven that miR-144-3p and ZEB1 in exosomes can regulate the development of OS by modulating the ferroptosis mechanism, and there are several advantages: First, we applied the CDTX model in vivo experiment which can provide an approximate simulation to the physiological conditions, thereby presenting a conclusion with solid clinical value; secondly, we collected exosomes extracted from OS tissues for sequencing. Compared with serum exosomes, exosomes from local tumor tissues can more accurately reflect the composition of the tumor microenvironment, which is convenient for further research on the communication between tumor cells and the impact on other cells around; third, we upregulated and knocked down miR-144-3p and ZEB1, respectively, sufficiently verified multiple possible pathways of ferroptosis, and conducted a functional rescue experiment to validate the function of miR-144-3p/ZEB1 axis on ferroptosis mechanism. The logic was strict and the evidence was sufficient, which makes our research results more convincing. In conclusion, these findings are helpful to further understand the mechanism of OS and confirm that ferroptosis-related miRNAs may have great potential as an anti-cancer therapeutic target or a diagnostic biomarker. In addition, with further study, the exosome may not only be used as a prognostic marker, but also as a therapeutic tool for the treatment of OS, all of which requires continuous research and exploration.

However, our research also has some limitations. For example, the sample scale was relatively small. It is necessary to further collect OS samples to verify the accuracy of this study. Some other clinical features have not been verified, such as tumor staging, and have not been tried in humans. It cannot be determined whether the results will be the same in human OS. At the same time, it is unknown whether exosomal therapy can be used for clinical treatment and whether it has serious side effects, all of which requires a lot of clinical research. In conclusion, more relevant studies should be carried out to further reveal and elaborate the relationship between miRNAs, ferroptosis, and OS, which has the potential to improve the treatment of OS patients.

## Supplementary Information

**Additional file 1: Supplementary Figure 1.** The whole uncropped images of the original WB of GPX4.

**Additional file 2: Supplementary Figure 2.** The whole uncropped images of the original WB of ACSL4.

**Additional file 3: Supplementary Figure 3.** The whole uncropped images of the original WB of xCT.

**Additional file 4: Supplementary Figure 4.** The whole uncropped images of the original WB of calnexin.

**Additional file 5: Supplementary Figure 5.** The whole uncropped images of the original WB of CD63.

**Additional file 6: Supplementary Figure 6.** The whole uncropped images of the original WB of HSP70.

**Additional file 7: Supplementary Figure 7.** The whole uncropped images of the original WB of TSG101.

**Additional file 8: Supplementary Table 1.** Primers, antibodies, Lentivirus (LV) and adenovirus (ADV).

**Additional file 9: Supplementary Table 2.** The diameter of gross specimens collected from CDTX.

## Data Availability

The data used to support the findings of this study are included in the article.
