# Supplementary figures and images for: Exosome-mediated miR-144-3p promotes ferroptosis to inhibit osteosarcoma proliferation, migration, and invasion through regulating ZEB1
Source: Mol Cancer. 2023 Jul 17;22:113. doi: 10.1186/s12943-023-01804-z (PMC10351131; doi:10.1186/s12943-023-01804-z)

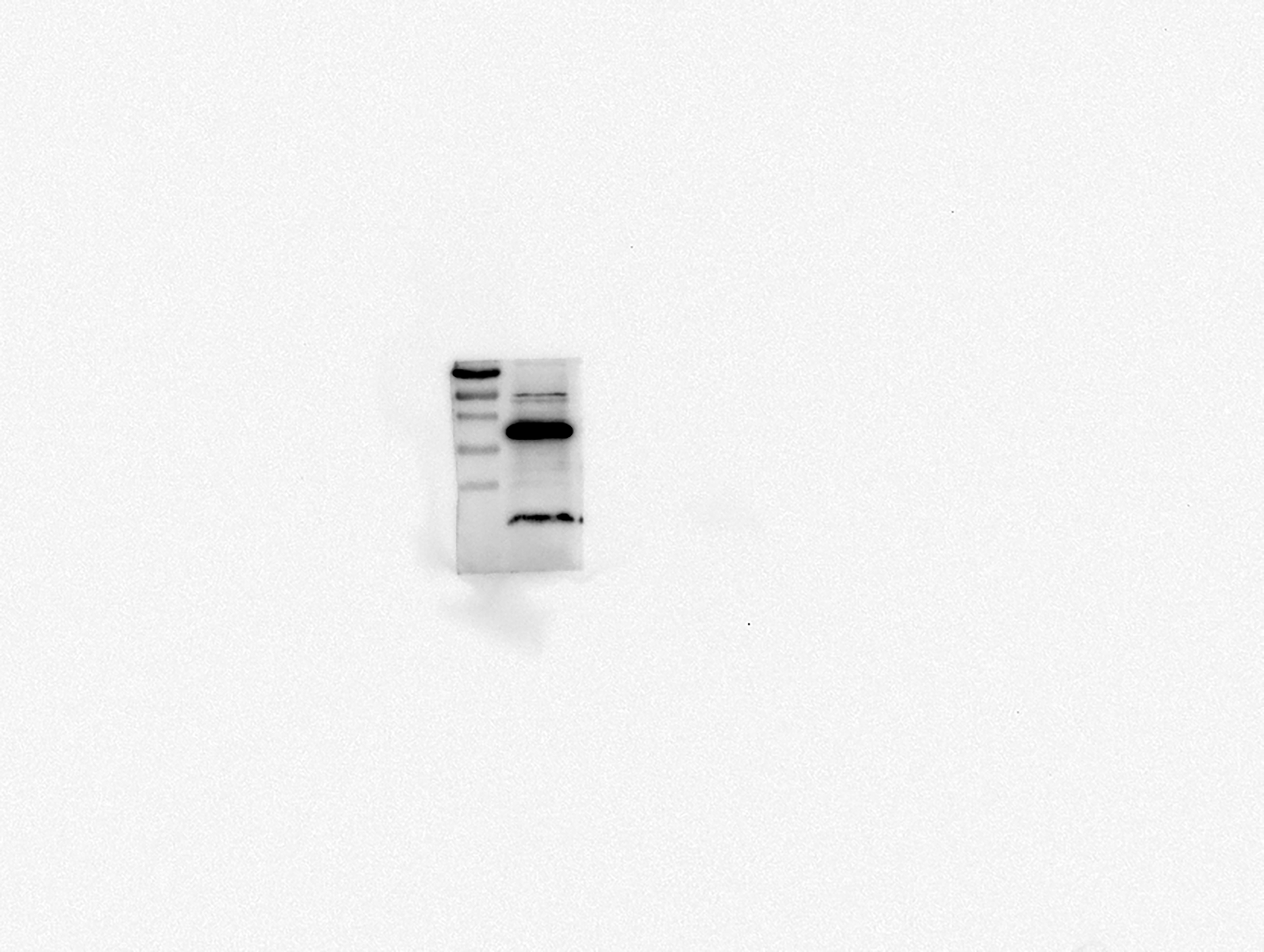

Supplement: Supplementary file 4 — Additional file 4: Supplementary Figure 4. The whole uncropped images of the original WB of calnexin. [file 12943_2023_1804_MOESM4_ESM.png]

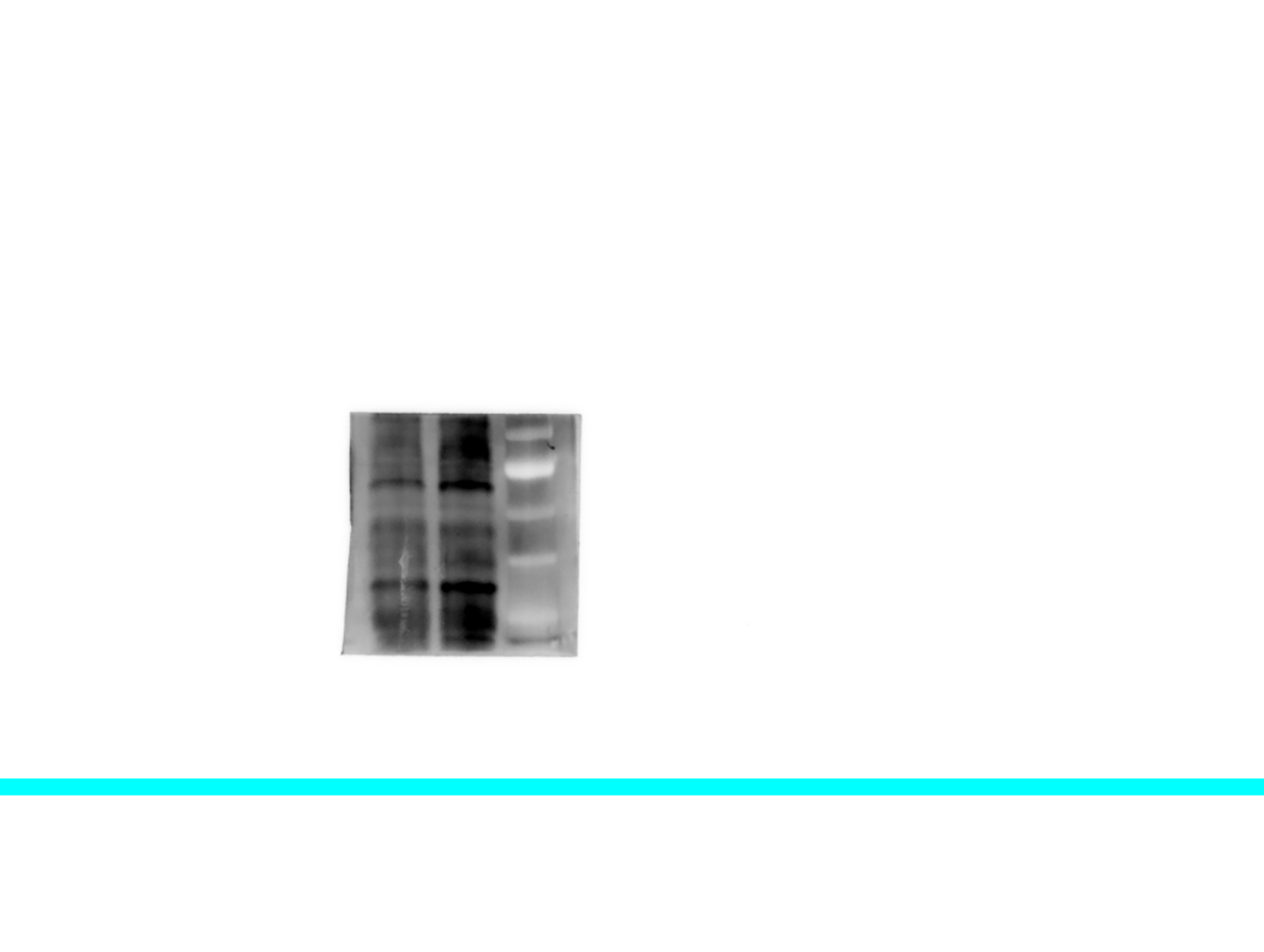

Supplement: Supplementary file 5 — Additional file 5: Supplementary Figure 5. The whole uncropped images of the original WB of CD63. [file 12943_2023_1804_MOESM5_ESM.png]

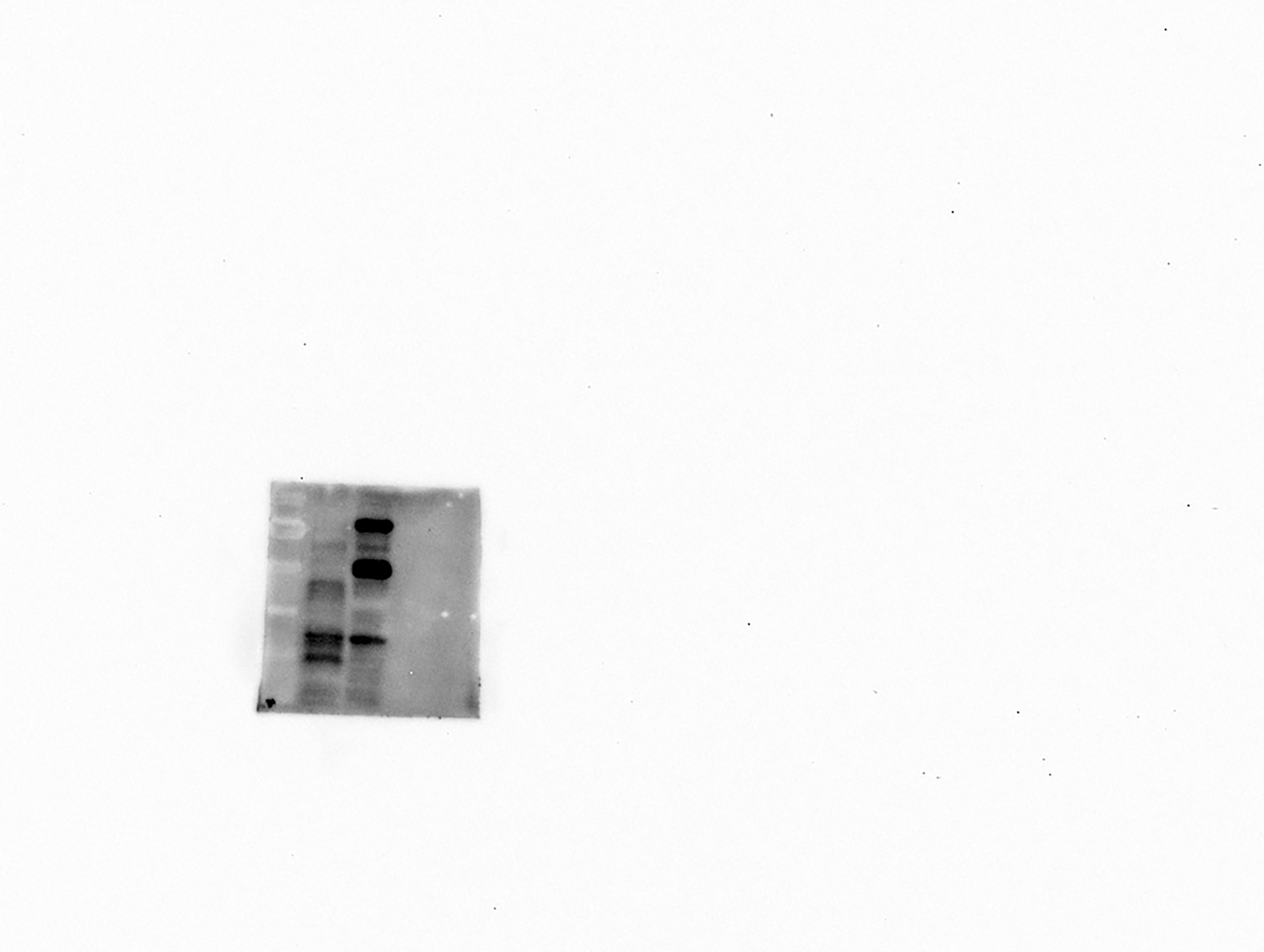

Supplement: Supplementary file 6 — Additional file 6: Supplementary Figure 6. The whole uncropped images of the original WB of HSP70. [file 12943_2023_1804_MOESM6_ESM.png]

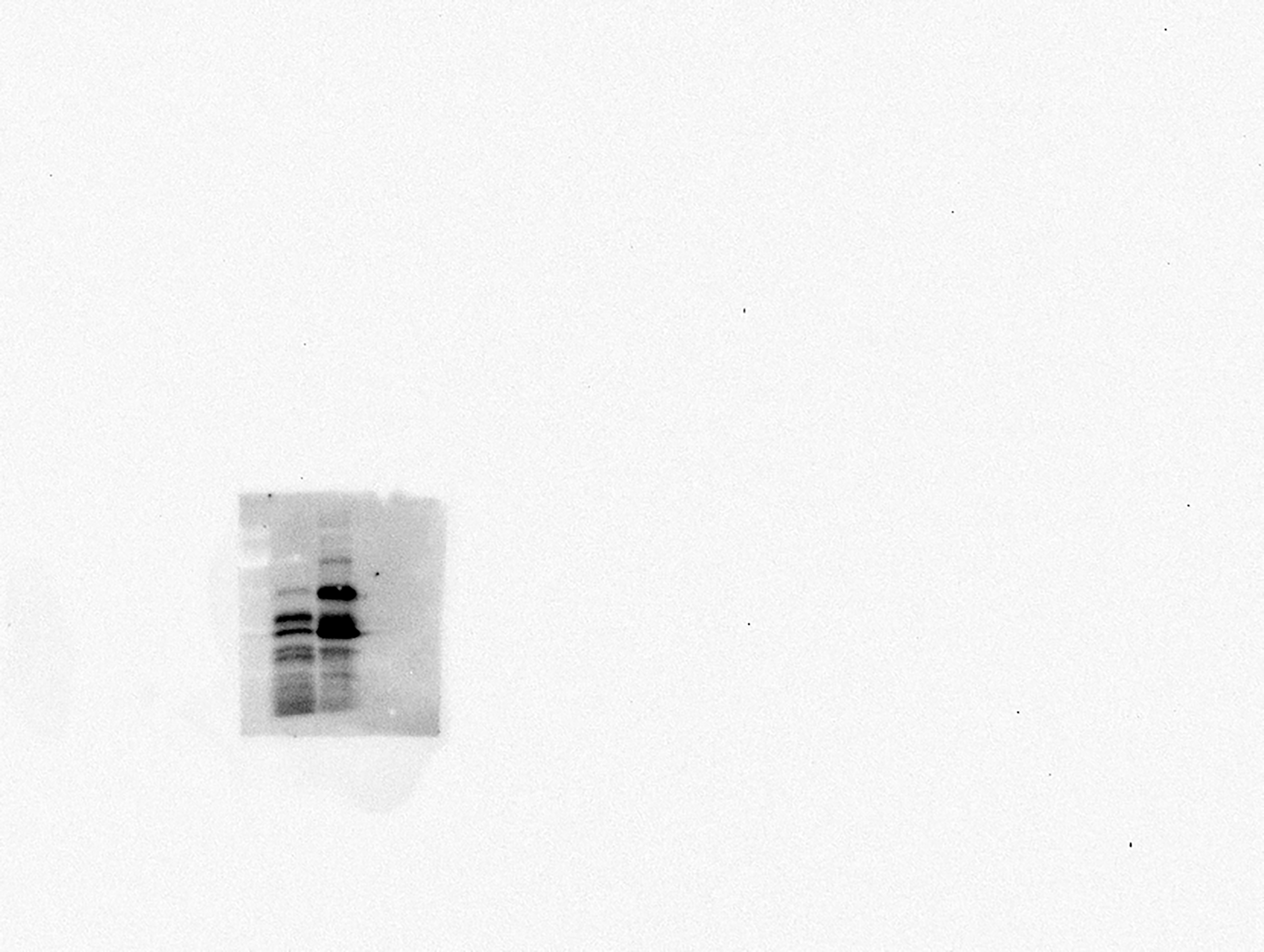

Supplement: Supplementary file 7 — Additional file 7: Supplementary Figure 7. The whole uncropped images of the original WB of TSG101. [file 12943_2023_1804_MOESM7_ESM.png]
