# Supplementary material for: Exosome-mediated miR-144-3p promotes ferroptosis to inhibit osteosarcoma proliferation, migration, and invasion through regulating ZEB1
Source: Mol Cancer. 2023 Jul 17;22:113. doi: 10.1186/s12943-023-01804-z (PMC10351131; doi:10.1186/s12943-023-01804-z)
Supplement: Supplementary file 8 — Additional file 8: Supplementary Table 1. Primers, antibodies, Lentivirus (LV) and adenovirus (ADV). [file 12943_2023_1804_MOESM8_ESM.docx]

**Supplementary Table 1. Primers, antibodies, Lentivirus (LV) and adenovirus (ADV).**

**Primer:**

miR144-3P-F (hsa) GCGCCGTACAGTATAGATGATGTACT (Nanning GenSys biotechnology, CN)

miR144-3P-R (hsa) was contained within the miRNA First Strand cDNA Synthesis kit (Tailing Reaction) (Sangon Biotech, CN).

miR144-3P-RT (hsa) was contained within the miRNA First Strand cDNA Synthesis kit (Tailing Reaction) (Sangon Biotech, CN).

ZEB1 (hsa) Fwd-5’AGTGTTACCAGGGAGGAGCAGTG-3’ (Sangon Biotech, CN)

ZEB1 (hsa) Rev-5’TTTCTTGCCCTTCCTTTCCTGTGTC-3’ (Sangon Biotech, CN)

GPX4 (hsa) Fwd-5’TGCTCTGTGGGGCTCTGG-3’ (Nanning GenSys biotechnology, CN)

GPX4 (hsa) Rev-5’GATGTCCTTGGCGGAAAACT-3’ (Nanning GenSys biotechnology, CN)

ACSL4 (hsa) Fwd-5’CCGACCTAAGGGAGTGATGAT-3’ (Nanning GenSys biotechnology, CN)

ACSL4 (hsa) Rev-5’CAGAGAGTGTAAGCGGAGAAGAA-3’ (Nanning GenSys biotechnology, CN)

xCT (hsa) Fwd-5’TCCCCCGTGTGTCCCTAC-3’ (Nanning GenSys biotechnology, CN)

xCT (hsa) Rev-5’TGATAATGGAGACTCCCCTCAG-3’ (Nanning GenSys biotechnology, CN)

**Antibody:**

| ZEB1 (IHC) | Primary antibody: 1:1000, Source: Rabbit, GB11513, Medikabazaar, Mumbai, India |
| --- | --- |
| ZEB1 (IHC) | Secondary antibody: Goat Anti-Rabbit IgG H&L, 1:5000, ab205718, abcam, Cambridge, UK |
| CD63 (WB) | Primary antibody: 1:700, Source: Rabbit, product number: bsm-52384R, Bioss, Beijing, CN |
| CD63 (WB) | Secondary antibody: Goat Anti-Rabbit IgG H&L, 1:5000, ab205718, abcam, Cambridge, UK |
| Calnexin (WB) | Primary antibody: 1:20000, Source: Rabbit, catalog number: 10427-2-AP, Proteintech Group, IL, USA |
| Calnexin (WB) | Secondary antibody: Goat Anti-Rabbit IgG H&L, 1:5000, ab205718, abcam, Cambridge, UK |
| TSG101 (WB) | Primary antibody: 1:8000, Source: Rabbit, catalog number: 28283-1-AP, Proteintech Group, IL, USA |
| TSG101 (WB) | Secondary antibody: Goat Anti-Rabbit IgG H&L, 1:5000, ab205718, abcam, Cambridge, UK |
| HSP70 (WB) | Primary antibody: 1:10000, Source: Rabbit, catalog number: 10995-1-AP, Proteintech Group, IL, USA |
| HSP70 (WB) | Secondary antibody: Goat Anti-Rabbit IgG H&L, 1:5000, ab205718, abcam, Cambridge, UK |
| ACSL4 (WB) | Primary antibody: 1:5000, Source: Mouse, catalog number: 66617-1-Ig, Proteintech Group, IL, USA |
| ACSL4 (WB) | Secondary antibody: 1:5000, Goat Anti-Mouse IgG (H+L), Catalog number: SA00001-1, Proteintech Group, IL, USA |
| GPX4 (WB) | Primary antibody: 1:3000, Source: Mouse, catalog number: 67763-1-Ig, Proteintech Group, IL, USA |
| GPX4 (WB) | Secondary antibody: 1:5000, Goat Anti-Mouse IgG (H+L), Catalog number: SA00001-1, Proteintech Group, IL, USA |
| xCT (WB) | Primary antibody: 1:3000, Source: Rabbit, datasheet: TA385257, OriGene, MD, USA |
| xCT (WB) | Secondary antibody: Goat Anti-Rabbit IgG H&L, 1:5000, ab205718, abcam, Cambridge, UK |
| ACSL4 (IHC) | Primary antibody: 1:800, Source: Mouse, catalog number: 66617-1-Ig, Proteintech Group, IL, USA |
| ACSL4 (IHC) | Secondary antibody: Rabbit Anti-Mouse IgG H&L, 1:3000, ab6728, abcam, Cambridge, UK |
| GPX4 (IHC) | Primary antibody: 1:3000, Source: Mouse, catalog number: 67763-1-Ig, Proteintech Group, IL, USA |
| GPX4 (IHC) | Secondary antibody: Rabbit Anti-Mouse IgG H&L, 1:3000, ab6728, abcam, Cambridge, UK |
| xCT (IHC) | Primary antibody: 1:300, Source: Rabbit, catalog number: 26864-1-AP, Proteintech Group, IL, USA |
| xCT (IHC) | Secondary antibody: Goat Anti-Rabbit IgG H&L, 1:5000, ab205718, abcam, Cambridge, UK |
| GAPDH (WB) | Primary antibody: 1:100000, Source: Mouse, catalog number: 60004-1-Ig, Proteintech Group, IL, USA |
| GAPDH (WB) | Secondary antibody: 1:5000, Goat Anti-Mouse IgG (H+L), Catalog number: SA00001-1, Proteintech Group, IL, USA |

**Lentivirus (LV) and adenovirus (ADV):**

The following LV and ADV were provided by Hanheng Biotechnology (Shanghai, CN)

LV-pre-miRNA

LV-sponge-miRNA

ADV-mRNA

LV-short hairpin (sh)-mRNA
LV-pre-miRNA-NC

LV-sponge-miRNA-NC

ADV-mRNA-NC

LV-sh-mRNA-NC
