# Supplementary material for: Exosome-mediated miR-144-3p promotes ferroptosis to inhibit osteosarcoma proliferation, migration, and invasion through regulating ZEB1
Source: Mol Cancer. 2023 Jul 17;22:113. doi: 10.1186/s12943-023-01804-z (PMC10351131; doi:10.1186/s12943-023-01804-z)
Supplement: Supplementary file 9 — Additional file 9: Supplementary Table 2. The diameter of gross specimens collected from CDTX. [file 12943_2023_1804_MOESM9_ESM.docx]

| **Supplementary Table 2. The diameter of gross specimens collected from CDTX** | | | | | | |
| --- | --- | --- | --- | --- | --- | --- |
|  | Replicate 1（cm^3^） | Replicate 2（cm^3^） | Replicate 3（cm^3^） | Mean（cm^3^） | Standard Deviation（cm^3^） | P |
| Group Ⅰ | 1.58 | 1.63 | 1.83 | 1.68 | 0.11 | 0.031 |
| Group Ⅱ | 1.42 | 1.34 | 1.45 | 1.40 | 0.05 |  |
| Group Ⅲ | 1.55 | 1.65 | 1.78 | 1.66 | 0.09 | 0.013 |
| Group Ⅳ | 1.99 | 1.96 | 2.14 | 2.03 | 0.08 |  |
| Group Ⅴ | 1.56 | 1.66 | 1.67 | 1.63 | 0.05 | 0.003 |
| Group Ⅵ | 2.01 | 2.24 | 2.20 | 2.15 | 0.10 |  |
| Group Ⅶ | 1.67 | 1.64 | 1.60 | 1.64 | 0.03 | 0.001 |
| Group Ⅷ | 0.95 | 1.12 | 1.01 | 1.03 | 0.07 |  |
| Group Ⅸ | 1.61 | 1.65 | 1.76 | 1.67 | 0.06 | 0.000 |
| Group Ⅹ | 1.46 | 1.50 | 1.42 | 1.46 | 0.03 |  |
| Group XI | 2.01 | 1.98 | 2.05 | 2.01 | 0.03 |  |
| Group XII | 1.74 | 1.72 | 1.80 | 1.75 | 0.03 |  |
